# Microbial Water Quality through a Full-Scale Advanced Wastewater Treatment Demonstration Facility

**DOI:** 10.1021/acsestengg.2c00198

**Published:** 2022-10-06

**Authors:** Scott Miller, Hannah Greenwald, Lauren C. Kennedy, Rose S. Kantor, Renjing Jiang, Aleksey Pisarenko, Elise Chen, Kara L. Nelson

**Affiliations:** † Department of Civil and Environmental Engineering, College of Engineering, University of California, Berkeley, Berkeley, California 94720, United States; ‡ National Science Foundation Engineering Research Center for Re-inventing the Nation’s Urban Water Infrastructure (ReNUWIt), Berkeley, California 94720, United States; § Department of Civil and Environmental Engineering, College of Engineering, Stanford University, Stanford, California 94305, United States; ∥ Trussell Technologies, Inc., Solana Beach, California 92075, United States

**Keywords:** potable reuse, treatment process monitoring, flow cytometry, enteric viruses, antibiotic resistance genes

## Abstract

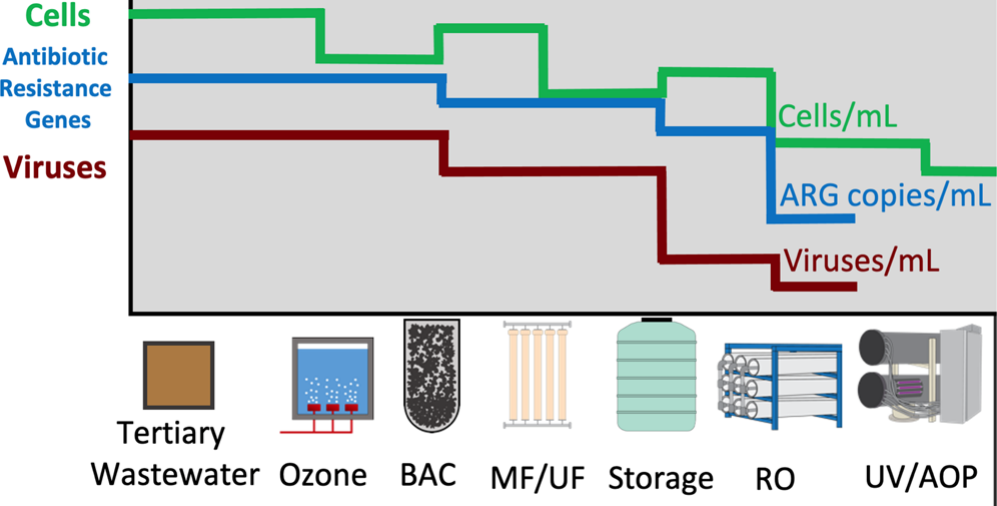

The fates of viruses, bacteria, and antibiotic resistance genes during advanced wastewater treatment are important to assess for implementation of potable reuse systems. Here, a full-scale advanced wastewater treatment demonstration facility (ozone, biological activated carbon filtration, micro/ultrafiltration, reverse osmosis, and advanced oxidation) was sampled over three months. Atypically, no disinfectant residual was applied before the microfiltration step. Microbial cell concentrations and viability were assessed via flow cytometry and adenosine triphosphate (ATP). Concentrations of bacteria (16S rRNA gene), viruses (human adenovirus and JC polyomavirus), and antibiotic resistance genes (*sul1* and *bla*_*TEM*_) were assessed via quantitative PCR following the concentration of large sample volumes by dead-end ultrafiltration. In all membrane filtration permeates, microbial concentrations were higher than previously reported for chloraminated membranes, and log_10_ reduction values were lower than expected. Concentrations of 16S rRNA and *sul1* genes were reduced by treatment but remained quantifiable in reverse osmosis permeate. It is unclear whether *sul1* in the RO permeate was from the passage of resistance genes or new growth of microorganisms, but the concentrations were on the low end of those reported for conventional drinking water distribution systems. Adenovirus, JC polyomavirus, and *bla*_*TEM*_ genes were reduced below the limit of detection (∼10^–2^ gene copies per mL) by microfiltration. The results provide insights into how treatment train design and operation choices affect microbial water quality as well as the use of flow cytometry and ATP for online monitoring and process control.

## 1 Introduction

Potable reuse provides an alternative source of drinking water to many municipalities around the world through the advanced treatment of wastewater.^1^ Advanced treatment processes can provide effective barriers to pathogenic microorganisms; because of the high levels of treatment, they also present unique challenges for quantifying microbial targets in treatment process effluents. For example, the concentrations of microorganisms in reverse osmosis (RO) permeate can be extremely low,^2^ requiring the concentration of large sample volumes to achieve low detection limits.^3^ In turn, sample concentration methods vary widely in recovery efficiencies,^4−7^ which must be known to accurately quantify microbial concentrations. To overcome these challenges, we used enhanced sampling and analytical techniques to better characterize the pathogenic viruses, emerging microbial contaminants (e.g., antibiotic resistance genes), and bacterial abundance and viability throughout an advanced treatment train used for potable reuse.

As an acute public health risk, pathogenic enteric viruses are often a primary driver in potable reuse regulation and design.^8^ Evaluating virus removal by advanced treatment trains is difficult because influent virus concentrations are often too low to demonstrate high removal, and there are no widely accepted surrogate or model organisms.^6,9−11^ Human adenovirus and JC polyomavirus have been proposed as model organisms for evaluating virus removal by wastewater treatment processes due to their relatively high prevalence, concentrations, and stability in raw wastewater.^12,13^ However, other studies report concentrations of these viruses below detection limits in conventional wastewater effluents.^10,14^ To improve the likelihood of detecting enteric viruses throughout advanced treatment (e.g., RO permeate), we used enhanced sampling methods (i.e., dead-end ultrafiltration) to concentrate large sample volumes to quantify enteric viruses.^4,5,15^

Regarding bacteria, research and regulation have largely focused on bacterial indicators (e.g., *E. coli*) or specific pathogens (e.g., *Salmonella enterica*),^16,17^ but broader evaluations of the bacterial community can yield insights into treatment performance and impacts that may be overlooked by current regulatory and design approaches.^18−20^ For example, antibiotic resistance genes and bacteria are ubiquitous in wastewater systems around the world^21^ but are generally not regulated.^3^ Further research on the removal and proliferation of resistance genes and bacteria in advanced treatment trains is needed to inform risk assessments for reuse systems.^22,23^ Some resistance genes, such as *sul1* and *bla*_*TEM*_ (encoding for resistance to sulfonamide drugs and β-lactam drugs, respectively), are often abundant in raw and conventionally treated wastewater and may be suitable indicators for removal of antibiotic resistance overall,^21,24^ but quantification of resistance genes in full-scale advanced treatment trains remains limited.^18,25−27^ Separately, quantification of genomic targets in the RO permeate requires a sample concentration method to reduce assay detection limits and overcome contamination.^18,19,25^ Ultrafiltration methods have been used effectively to concentrate microbial biomass from ground and surface waters^5,15^ and drinking waters,^4^ but we found no reports of their use on advanced treatment effluents. Therefore, we report the first evaluation of dead-end ultrafiltration to recover cells from diverse treatment process effluents (i.e., tertiary wastewater to RO permeate) at an advanced treatment facility.

Improved analytical methods for quantification of cells (e.g., flow cytometry and ATP analysis) could also be harnessed to enhance monitoring and validation of treatment process performance. For example, RO membranes are often awarded pathogen removal credits equal to the measured reduction of a continuously monitored surrogate,^3^ but the removal of conventional surrogates (e.g., total organic carbon) is typically low (approximately 1–2 log_10_).^28^ In contrast, ATP removal by RO can exceed 3 log_10_,^29,30^ and recent commercialized products can provide continuous measurement of ATP.^30^ Furthermore, as systems seek to maximize the benefits of treatments such as biological activated carbon (BAC) filtration, the use of flow cytometry and/or ATP may serve as a relatively accurate,^31^ affordable,^32^ and sensitive^33^ approach for assessing biological activity and performance in full-scale facilities.

The objectives of this study were to investigate, at a full-scale advanced wastewater treatment demonstration facility, the effects of advanced treatment processes on the concentrations of two enteric virus gene targets (human adenovirus and JC polyomavirus) and two antibiotic resistance genes (*sul1* and *bla*_*TEM*_), as well as microbial abundance and viability (cell counts, ATP concentrations, 16S rRNA gene counts). We also evaluated dead-end ultrafiltration for concentrating large volumes of water after each treatment step (up to 4000 L for RO permeate) and quantified the recovery efficiency using cell counts. Sampling was conducted at an advanced treatment facility that was operating without any disinfectant residual (often applied to mitigate fouling), which offered a unique opportunity to study each unit process without the confounding effect of a disinfectant. This research addressed two ongoing needs for the implementation of advanced treatment for potable reuse: monitoring strategies for crediting pathogen reduction and improved process control. The results provide insights into how treatment train design and operation choices affect microbial water quality as well as the use of flow cytometry and ATP for online monitoring.

## 2 Study Site, Methods, and Materials

### 2.1 Layout and Operation of the Advanced Treatment Facility

**Figure 1:**
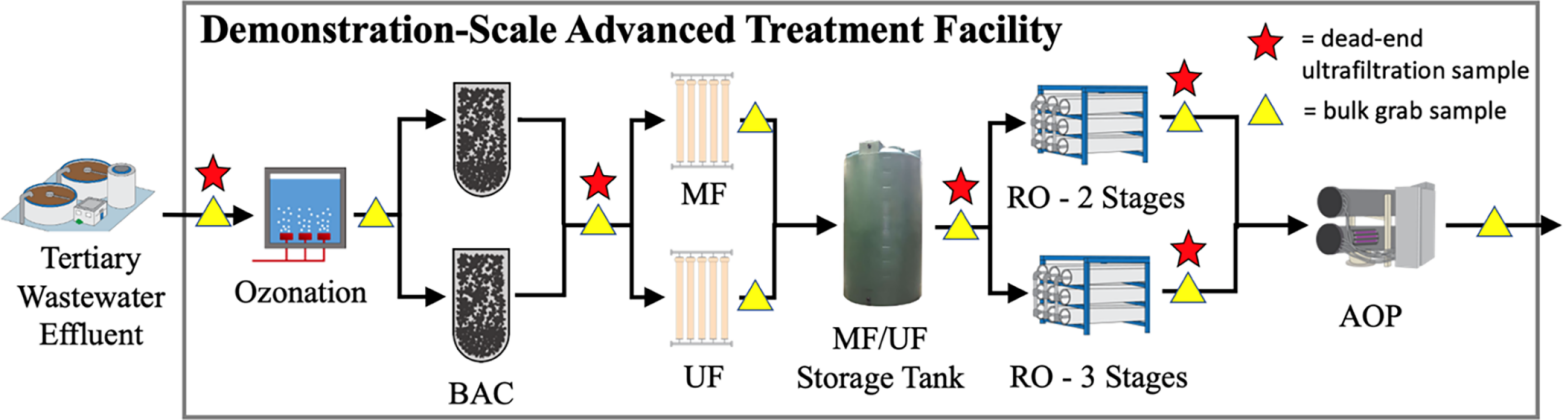
Schematic of the advanced treatment train. Unchlorinated tertiary wastewater effluent was treated sequentially by ozonation, parallel biologically activated carbon (BAC) filtration, parallel microfiltration (MF) and ultrafiltration (UF), parallel reverse osmosis units with two or three stages in series (RO), and an ultraviolet–free chlorine advanced oxidation process (AOP).

We sampled a full-scale advanced wastewater treatment demonstration facility intended for potable reuse in the United States with a treatment capacity of ∼3.8 × 10^6^ L/day. The advanced wastewater treatment facility was designed to test the performance of several different parallel treatment processes, as illustrated in Figure 1. Raw wastewater was treated at a conventional wastewater treatment facility, which included primary (sedimentation), secondary (activated sludge to achieve complete nitrification), and tertiary treatment processes (granular media filtration, 7 feet of 1.0 mm anthracite coal). Unchlorinated tertiary effluent was then treated sequentially at the advanced wastewater treatment facility during the time of study by (i) ozonation (Wedeco/Xylem), with an average target applied ozone concentration of 8.17 mg/L, CT range of 1.84–4.00 (average = 2.68) mg min/L, and hydraulic retention time of ∼7.5 min to target 1 log_10_ reduction of *Cryptosporidium*, where ozone CT was calculated using a modified extended integration CT method as previously described;^34^ (ii) two parallel, identical biologically activated carbon filters (“BAC”, Leopold/Xylem), each containing granular activated carbon media and operated with empty bed contact times of 15 min to achieve a filter effluent turbidity of less than 0.3 NTU and average backwash intervals of approximately two to four days; (iii) parallel microfiltration (“MF”, Pall Corporation) and ultrafiltration (“UF”, modules by Toray, and overall system design by H_2_O Innovation) membranes, with nominal pore sizes of 0.1 and 0.015 μm, respectively, filter fluxes of 30 and 60 gallons per square foot per day, respectively, average water recoveries of 96% each, backwash intervals of 30 min, and daily pressure decay tests to evaluate membrane integrity; (iv) a storage tank (MF/UF storage tank) with a hydraulic retention time of approximately 30 min when both RO trains were online; (v) two parallel reverse osmosis (“RO”, EnAqua) units, one with two stages in series and the other with three stages in series, each operated at an average water recovery of 75–80%; and (vi) a UV and free chlorine advanced oxidation process (“AOP”, TrojanUV), with free chlorine concentrations in the feed and effluent of approximately 2 and 1–1.5 mg/L as Cl_2_, respectively, a hydraulic retention time of approximately 15 s, and a minimum UV dose of 850 mJ/cm^2^ that achieved an average 1.70 log_10_ reduction of 1,4-dioxane.^2^

During our sample collection period, the facility was utilized as a testbed to investigate the impacts of reduced chloramine use on membrane fouling and AOP performance. Until the start of this study period, a chloramine residual of approximately 1–2 mg/L as Cl_2_ was applied upstream of MF/UF to reduce membrane biofouling. However, no chloramine was applied from September 1 to December 31, 2017, which included the full duration of this study.

### 2.2 Bulk Water Collection via Grab Sampling and Concentration by Dead-End Ultrafiltration

We sampled major treatment process effluents (see Figure 1) between September 14, 2017, and December 14, 2017 (Table S1). Sample taps were wiped with an ethanol towelette and allowed to air dry. For all types of sample collection, sample taps were flushed for the following durations prior to collection of bulk water: tertiary wastewater, ozone, and BAC (>5 min), MF, UF, and MF/UF storage tank (>15 min), and RO and AOP (>30 min). Sample dates, times, and the total number of grab samples and samples for dead-end ultrafiltration are presented in Table S1. Bottles for flow cytometry and ATP grab samples (500 mL) were rinsed with sample water three times prior to collection and quenched (if necessary) with excess sodium thiosulfate. Flow cytometry and ATP samples were transported on ice to the laboratory and maintained at 4 °C until further processing. Collection of operational data and sampling for physical and chemical water quality parameters (i.e., chloramine and ozone residuals, turbidity) were conducted by our project partners.

Water was concentrated by dead-end ultrafiltration for quantitative polymerase chain reaction (qPCR) from tertiary wastewater, BAC filtrate, MF/UF storage tank, and RO permeate (Figure 1). The primary concentration of bulk water microbial biomass was conducted using dead-end ultrafiltration as previously described,^4^ with one modification of overnight blocking of ultrafilters (REXEED 25S, Henry Schein, Melville, NY) with 5% w/v sterile-filtered bovine calf serum (catalog #12133C, Fisher Scientific) prior to use. The use of filter blocking was based on previous studies^5,35^ that used blocked ultrafilters on drinking and surface waters to achieve high recoveries of various bacteria and viruses.^5,36^ Filters were rinsed of blocking solution with deionized water prior to assembly and used for sample filtration. Filtration volumes varied by the sample location (range: 30 L for tertiary wastewater to 4000 L for RO permeate) to collect as much biomass as possible while avoiding filter clogging. Detailed information on volumes of water collected, sample water turbidity, and recovery efficiencies are presented in Table S12. After filtration, ultrafilters were transported on ice and stored at 4 °C for up to 3 h before backflushing. Backflushing consisted of pumping 500 mL of backflush solution (0.5% w/v Tween 80, 0.01% w/v sodium polyphosphate, and 0.001% w/v Y-30 antifoam emulsion) through the ultrafilter and into a sterile container in the opposite direction from sample filtering, as previously described.^4^

Secondary concentration to further concentrate ultrafilter backflush water was conducted using polyethylene glycol precipitation (PEG, i.e., flocculation and centrifugation).^37^ Briefly, 1.15% w/v NaCl, 8% w/v poly(ethylene glycol), and 1% w/v beef extract (catalog #DF0115173, Fisher Scientific) were added to the backflush water. The solution was vigorously stirred on a magnetic stirrer for 1 h at 4 °C, incubated at 4 °C overnight, and centrifuged into pellets at 3665 relative centrifugal force (Sorvall RC 5C with SH-3000 rotor; ThermoFisher Scientific) for 45 min at 4 °C. Pellets were resuspended in 1–4 mL of sterile tris-EDTA buffer and immediately stored at −80 °C until DNA extraction.

Field blanks for dead-end ultrafiltration field sampling were created by processing an ultrafilter alongside field samples, including filter blocking, backflushing, and secondary concentration. After overnight blocking, field blank ultrafilters were flushed with 1 L of autoclaved DI water (via crossflow filtration) to remove the blocking solution, capped with sterilized caps, brought to the field, retained at ambient temperature during sample filtration, and then returned to the laboratory for parallel processing with field samples.

The recovery efficiencies for primary and secondary concentration steps were calculated on a subset of ultrafiltration samples using total cell counts by flow cytometry. For primary recovery, the initial sample was collected prior to starting ultrafiltration, and the final sample was collected from the ultrafiltration backflush water. The ultrafiltration backflush also served as the initial sample for calculating secondary recovery. For secondary recovery, only RO permeate samples were analyzed because centrifuged pellets of other sample types could not be adequately dispersed. Prior to freezing pellets at −80 °C, RO sample pellets were resuspended via repeated gentle pipetting until the sample matrix appeared homogenous (at least 10 s), and a subsample was analyzed by flow cytometry. Equations for recovery efficiency calculations are provided in the Supplementary Information.

### 2.3 Cell Counts by Fluorescent Staining and Flow Cytometry

Total and intact cell counts were measured in triplicate on an Accuri C6 flow cytometer (BD Biosciences, San Jose, CA) as previously described^29^ but using analysis volumes of 1 or 1.5 mL. Determination of quantification limits was also presented previously.^29^ In a previous study, researchers using a similar flow cytometry method demonstrated that cell counts include both bacteria and archaea.^38^ We quantified high nucleic acid bacteria using a published template.^39^

Results for cell counts and ATP for each sampling location were non-normally distributed (*p* < 0.05; Shapiro–Wilk); therefore, these results are presented using geometric means and geometric standard deviations. The calculation of log_10_ reduction values for cell counts and ATP across individual advanced treatment processes was based on process feed and effluent data from each individual sampling day. All significance testing for log_10_ reductions utilized a Student’s *t*-test and compared the calculated log_10_ reduction values against zero, unless otherwise specified.

All boxplot graphs for flow cytometry, ATP, and qPCR data in the main manuscript and Supplemental Information display data as follows: the middle bolded horizontal line is the median; the lower and upper “hinges” correspond to the first and third quartiles (the 25th and 75th percentiles), respectively; the bottom and top of the vertical lines are the minimum and maximum, respectively; and any dots above and beyond the bottom and top vertical lines are outliers.

### 2.4 Measurement of Adenosine Triphosphate Concentrations

Total and intracellular ATP concentrations were measured in technical triplicate as previously described using BacTiter-Glo Microbial Cell Viability Assay reagents (G8231, Promega Corporation, Madison, WI) with a GloMax^R^ 20/20 luminometer (Turner BioSystems, Sunnyvale, CA).^29^ For statistical analyses and log_10_ reduction calculations, all values below the quantification limit were set at the quantification limit (1 × 10^–4^ and 1.82 × 10^–5^ nM for total and intracellular ATP, respectively), as previously described.^29^

### 2.5 DNA Extraction

Following PEG precipitation, DNA was extracted from sample pellets using a PowerSoil Pro extraction kit (Qiagen, Germantown, MD), according to the manufacturer protocol, with slight modifications. Briefly, pellets were thawed and homogenized by vigorous vortexing for 10 s. For tertiary influent, BAC, and MF/UF storage tank samples, 200 μL of homogenized pellet (of approximately 2 mL total pellet) was added directly to a PowerSoil Pro PowerBead tube. For RO samples, homogenized pellets were centrifuged (15,000*g* for 15 min). The supernatant was aspirated and aliquoted onto a centrifugal filtration unit (Amicon ultra-15 centrifugal filter unit, 100 kDa; Millipore, Cork, Ireland) and centrifuged (7500*g* for 30 min). The concentrated supernatant was used to resuspend the centrifuged pellet, and 200 μL of rehomogenized pellet was added to the PowerBead tube. All samples were incubated at 37 °C for 30 min in an enzymatic digestion solution: 50 μL of 0.001% lysozyme (Sigma-Aldrich, Darmstadt, Germany), 50 μL of 0.00001% achromopeptidase (Sigma-Aldrich, Darmstadt, Germany), and 8 μL of 0.01% carrier RNA in buffer AVL (Qiagen, Germantown, MD). Finally, 500 μL of solution CD1 (PowerSoil Pro) was added, and extraction followed the PowerSoil Pro manufacturer protocol. The elution buffer was incubated on the elution filter for 5 min at room temperature prior to the final elution step. The effective volume extracted was recorded for each sample and was immediately frozen at −80 °C until use.

### 2.6 Quantitative PCR

qPCR sequences for primers and probes were selected (unaltered) from the literature to target the 16S rRNA gene,^40^ human adenovirus,^41^ JC polyomavirus,^42^*bla*_*TEM*_,^43^ and *sul1*.^43^ Amplification and quantification of genes were carried out in technical triplicate in MicroAmp Fast Optical 96-well optical plates (catalog #4346906, ThermoFisher Scientific) on a StepOnePlus real-time PCR system (software v2.3; Applied Biosystems, Foster City, CA). DNA standard curves consisted of 10-fold serial dilutions of gBlocks Gene Fragments (Integrated DNA Technologies, Coralville, IA; see Supplementary Information and Table S2) ranging from 10 to 10^9^ gene copies, depending on the assay, using PCR-grade water (catalog #AAJ60610-EQC, VWR, ThermoFisher Scientific) in DNA LoBind 0.5 mL (catalog #22431005, Eppendorf, Millipore Sigma) or 5 mL tubes (catalog #Z768820-200EA, Sigma-Aldrich). Triplicate negative controls (i.e., PCR-grade water) were run on every plate.

Data analysis of qPCR results was completed in R (v4.1.3). Standard curves on each qPCR plate were used to calculate gene counts for samples on each respective plate (i.e., sample curves were not pooled). The limit of detection (LoD) for each assay was experimentally determined as the lowest concentration on standard curves that was statistically different from negative controls and for which at least 75% of all triplicates were amplified. The LoDs were determined to be 1000 gene copies per reaction for the 16S rRNA gene and 10 gene copies per PCR reaction for all other qPCR assays. For statistical analyses and log_10_ reduction calculations, all values below the LoD were set at the LoD. Further information on the standard curves and negative controls is provided in Supplementary Information Tables S3 and S4, and Figure S1. The negative controls for the 16S rRNA gene amplified but below the LoD (i.e., not within the linear region of the standard curves). Negative controls and field blanks for all other assays did not amplify (data not shown). Additional information on data analysis is provided in the Supplementary Information.

Thermal cycling conditions for each assay were based on previous studies (Table S5); for each assay, we optimized the temperature and duration for each thermal cycling step to maximize amplification efficiency and the number of replicates amplifying at the LoD before analysis of any samples. Melt curves (SYBR Green chemistry assays) were used to evaluate nontarget amplification and confirm amplification of target DNA (results not shown). Inhibition testing of samples (Table S6) followed the spike and dilute method to determine possible inhibition of qPCR assays by interfering substances in the water samples and subsequent need for sample dilution.^44^ Based on inhibition testing results, sample DNA was diluted as necessary to ensure that <100 ng of DNA was added to each well. Further details on inhibition testing and sample dilution are provided in the Supplementary Information.

Reactions (total volume: 20 μL) were performed manually in triplicate with purified sample DNA (5 μL) and a reaction mix (15 μL). Assays utilized either TaqMan Environmental Master Mix 2.0 chemistry (catalog #4396838, ThermoFisher Scientific) or PowerUp SYBR Green Master Mix (catalog #A25780, ThermoFisher Scientific). Each reaction mix (Table S5) consisted of a master mix (10 μL), bovine serum albumin to minimize potential inhibition (0.3 μM; catalog #15260037, ThermoFisher Scientific), primers and probes, and PCR water to yield 15 μL.

## 3 Results

### 3.1 Fate of Enteric Viruses through Advanced Treatment

**Figure 2:**
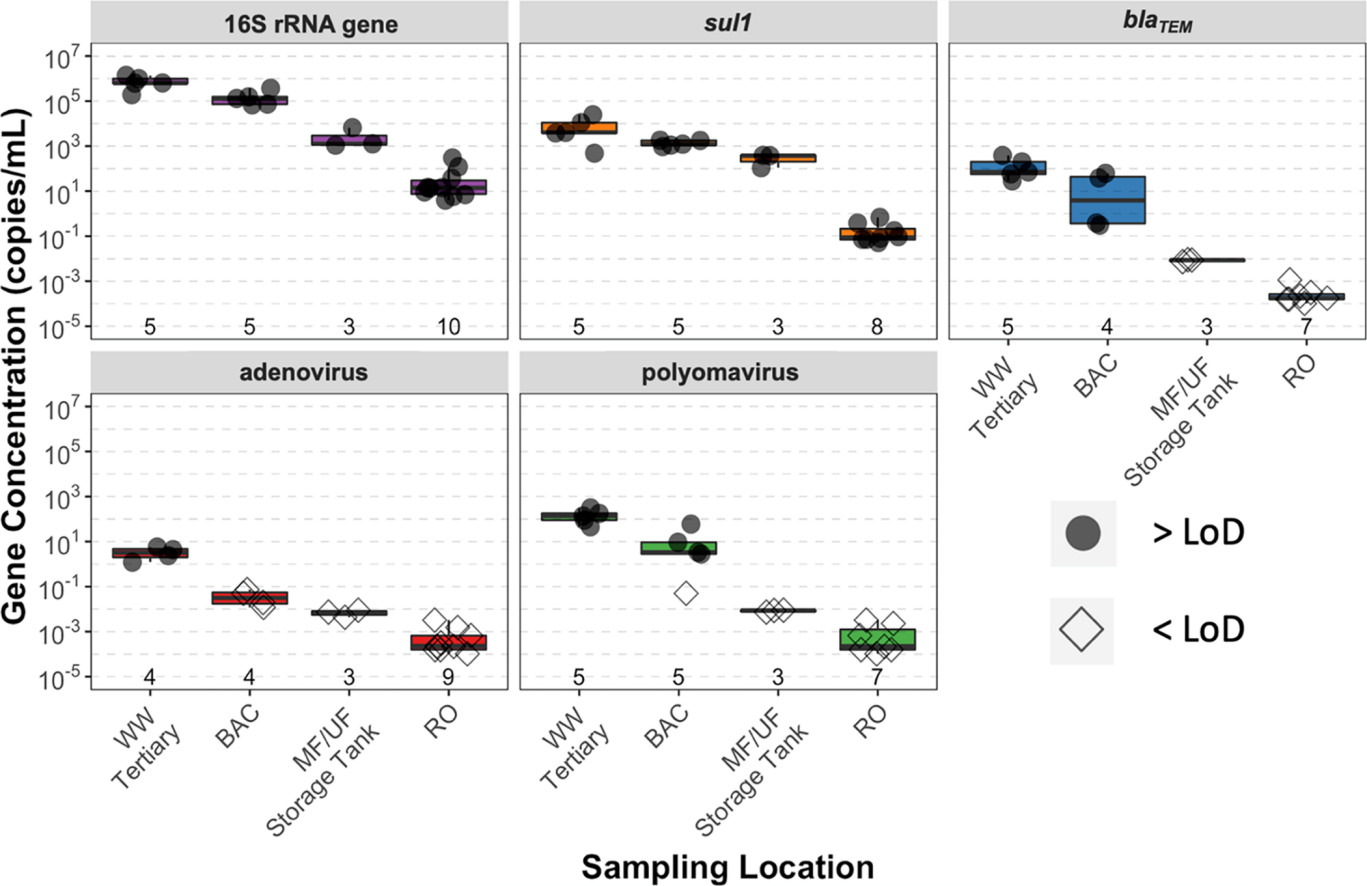
Boxplots of qPCR results for the 16S rRNA gene, two antibiotic resistance genes (*sul1* and *bla*_*TEM*_), and two enteric viruses (human adenovirus and JC polyomavirus). Shown immediately above the x-axis are the total number of samples for each qPCR assay at each sampling location. Data shown for RO include measurements from two parallel treatment processes. Results above the limit of detection (>LoD) are shown as open circles and results below the limit of detection (<LoD) are shown as open diamonds. Note that the LoD varied based on the volume of water filtered for each sampling event. All qPCR assays were carried out in technical triplicate. The apparent decreasing concentrations for *bla*_*TEM*_, adenovirus, and polyomavirus are due to the decrease in the LoD achieved by concentrating larger volumes of sample for each subsequent unit process; thus, these values represent a lower bound of removal. Sample numbers varied because there was insufficient extracted DNA to supply every qPCR assay.

Human adenovirus and JC polyomavirus gene targets were present in detectable concentrations in the tertiary wastewater, with geometric mean concentrations of 2.44 × 10^0^ and 1.06 × 10^2^ copies/mL, respectively. Despite these fairly high concentrations relative to previous reports^7,45−47^ and the use of large filtration volumes, concentrations of both viruses were reduced to below the limit of detection (LoD) after the BAC filter and after MF/UF, respectively (Figure 2, with summary statistics presented in Table S7). Through the combination of ozonation and BAC filtration, adenovirus and polyomavirus gene targets were reduced by >0.79 ± 0.40 and ≥1.45 ± 0.95 log_10_, respectively. In turn, the MF/UF reduced polyomavirus by >2.0 log_10_. The measured removals for adenovirus and polyomavirus by BAC filtration and MF/UF, respectively, are minimum values because samples from the respective process effluents did not amplify above the LoD. This sampling approach demonstrated that the advanced treatment train (from tertiary wastewater to RO permeate) provided at least 4 to 5 log_10_ cumulative reductions for polyomavirus and at least 2–3 log_10_ for adenovirus. It was possible to measure higher removal of polyomavirus (relative to adenovirus) because of the higher concentration of polyomavirus in the tertiary wastewater.

### 3.2 Fate of Antibiotic Resistance through Advanced Treatment

As expected, concentrations of 16S rRNA gene, *sul1*, and *bla*_TEM_ decreased at each sequential treatment step to low levels (gene concentration data in Figure 2, with summary statistics in Table S7). In the tertiary wastewater, geometric mean concentrations of the 16S rRNA gene, *sul1*, and *bla*_TEM,_ were 6.35 × 10^5^, 4.59 × 10^3^, and 9.74 × 10^1^ copies/mL, respectively. From tertiary wastewater to BAC filtration, average log_10_ reduction values for the 16S rRNA gene, *sul1*, and *bla*_*TEM*_ were 0.63, 0.63, and 1.23 log_10_, respectively. In the MF/UF storage tank and RO, all samples of the 16S rRNA gene and *sul1*, but no samples of *blaTEM*, were above the LoD. Average log_10_ reductions differed slightly for the 16S rRNA gene and *sul1* at the MF/UF (1.91 and 1.19 log_10_, respectively) and RO permeates (2.20 and 2.61 log_10_, respectively).

In the RO permeate, all samples amplified for the 16S rRNA gene and *sul1*, yielding geometric mean concentrations of 1.88 × 10^1^ and 1.07 × 10^–1^ copies/mL (Table S7), respectively. In turn, cumulative average log_10_ reductions by RO permeate for *sul1* and the 16S rRNA gene were similar at 4.37 and 4.59 log_10_, respectively, considering only sample days on which *sul1* and 16S rRNA gene count data were available for the tertiary wastewater and RO permeate (*n* = 3). Therefore, the geometric mean relative abundance of *sul1* (i.e., copies *sul1*/copies of 16S rRNA gene) was fairly similar between the tertiary wastewater (7.23 × 10^–3^) and RO permeate (9.60 × 10^–3^), with slightly higher relative abundances in the BAC (1.02 × 10^–2^) and MF/UF storage tank (5.53 × 10^–2^); however, none of the changes in *sul1* relative abundance at each treatment step were statistically significant (*p* > 0.05 for all, Student’s *t*-test).

### 3.3 Microbial Concentrations and Viability Throughout the Advanced Treatment Train

**Figure 3:**
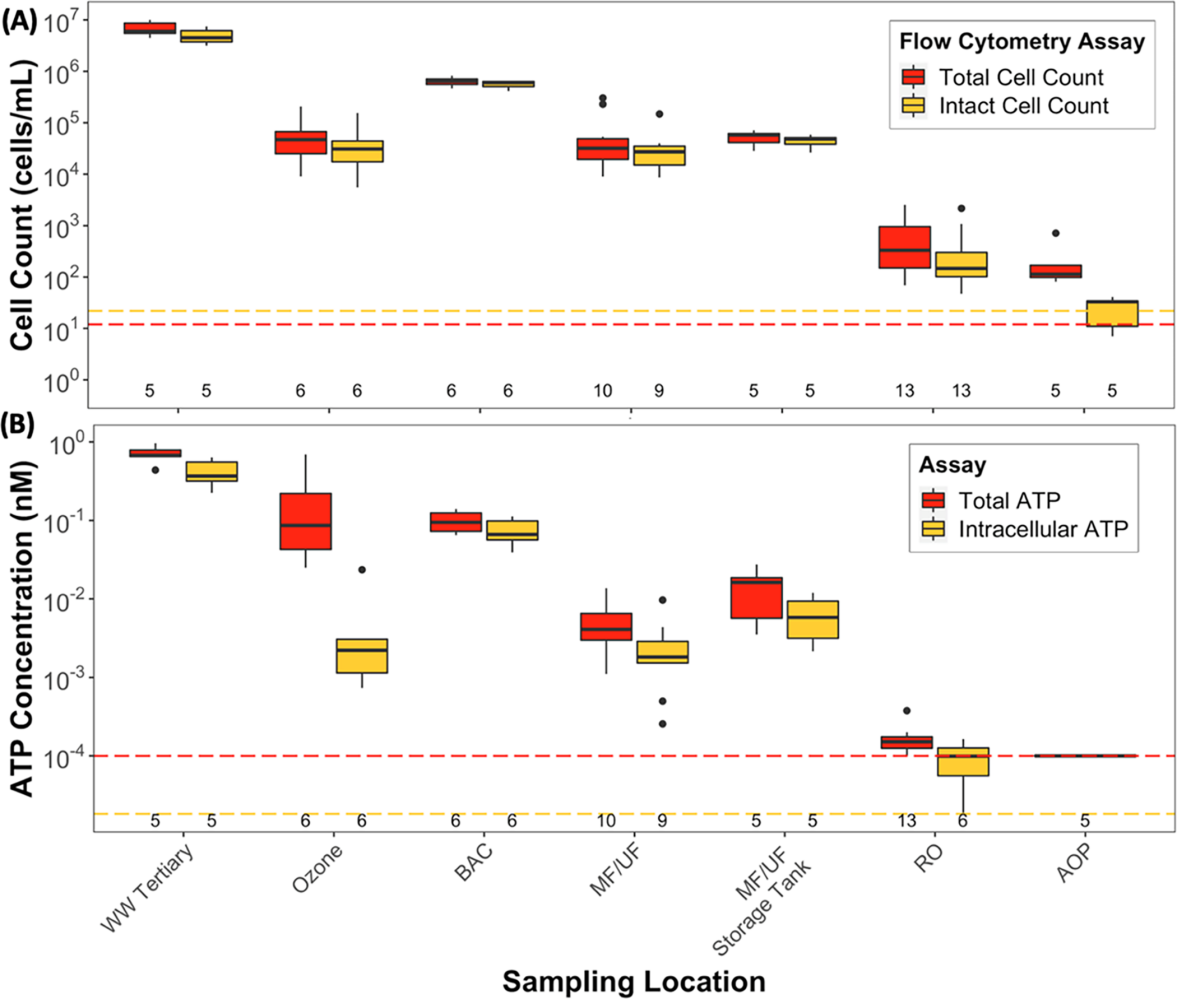
Boxplots of (A) total and intact cell counts and (B) total and intracellular ATP throughout the advanced treatment train. Data shown for MF/UF and RO include measurements from two parallel treatment processes. The lower limits of quantification for total and intact cell counts (12 and 22 cells/mL) and total and intracellular ATP (1 × 10^–4^ and 1.82 × 10^–5^ nM) are indicated by the red and yellow colored lines, respectively. The total number of samples taken (*n*) at each location is reported immediately above the *x*-axis. All samples were analyzed in technical triplicate. A complementary graph (Figure S2) showing log_10_ reduction values at each treatment step is available in the Supplementary Information. Sample numbers are lower for some sampling locations because, due to logistical challenges, we were unable to complete analyses on all samples on one sample day (October 10, 2017) and intracellular ATP analysis on RO permeate was discontinued after three sampling days to reduce daily sample processing time.

Concentrations of cells (by flow cytometry), ATP, and the 16S rRNA gene were strongly influenced by every major treatment process (Table 1, Figure 3a,b, Tables S8–S11). Log_10_ reduction values determined from total cell counts, total ATP, and 16S rRNA gene copies were generally similar for each treatment process (Table 1 and Figure S2). However, a few key differences among the microbial quantification methods were observed, and the use of different quantification methods to differentiate viable and nonviable cells provided insights into treatment performance.

**Table 1:** Summary of Bulk Water Results for the Five Assays Used to Quantify Microbial Counts Throughout the Advanced Treatment Train^a^

result	assay	units	tertiary wastewater	ozone	BAC	MF/UF	MF/UF storage tank	RO	AOP
geometric mean	total ATP	nM	6.86 × 10^–1^	1.05 × 10^–1^	9.50 × 10^–2^	4.42 × 10^–3^	1.11 × 10^–2^	1.54 × 10^–4^	1.00 × 10^–4^
	intracellular ATP	nM	3.96 × 10^–1^	2.56 × 10^–3^	6.99 × 10^–2^	1.81×10^–3^	5.37 × 10^-–3^	7.52 × 10^–5^	n.d.
	total cell count	cells/mL	6.63 × 10^6^	4.31 × 10^4^	6.28 × 10^5^	3.90 × 10^4^	4.95 × 10^4^	3.53 × 10^2^	1.61 × 10^2^
	intact cell count	cells/mL	4.77 × 10^6^	2.89 × 10^4^	5.57 × 10^5^	2.65 × 10^4^	4.30 × 10^4^	2.04 × 10^2^	2.95 × 10^1^
	16S rRNA gene	gene counts/mL	6.35 × 10^5^	n.d.	1.30 × 10^5^	n.d.	2.08 × 10^4^	1.88 × 10^1^	n.d.
									
geometric standard deviation (of the geometric mean)	total ATP		1.34	3.52	1.39	2.11	2.38	1.42	n.d.
	intracellular ATP		1.53	3.43	1.51	2.98	2.06	2.25	n.d.
	total cell count		1.40	2.93	1.23	3.08	1.45	3.32	2.40
	intact cell count		1.42	3.06	1.20	2.26	1.37	3.28	1.32
	16S rRNA gene		2.10	n.d.	1.99	n.d.	2.71	3.98	n.d.
									
log_10_ removal value	total ATP		n.d.	0.73	0.04	1.33*	–0.34	1.82*	0.44*
	intracellular ATP		n.d.	2.20*	–1.44*	1.59*	–0.36*	1.82*	n.d.
	total cell count		n.d.	2.23*	–1.16*	1.21*	–0.19	2.28*	0.39*
	intact cell count		n.d.	2.24*	–1.28*	1.32*	–0.12	2.21*	0.86*
	16S rRNA gene		n.d.	n.d.	n.d.	n.d.	n.d.	n.d.	n.d.
									
cumulative log_10_ removal value	total ATP		n.d.	n.d.	0.90	n.d.	n.d.	3.78	n.d.
	intracellular ATP		n.d.	n.d.	0.80	n.d.	n.d.	3.81	n.d.
	total cell count		n.d.	n.d.	0.99	n.d.	n.d.	4.04	n.d.
	intact cell count		n.d.	n.d.	0.90	n.d.	n.d.	4.16	n.d.
	16S rRNA gene		n.d.	n.d.	0.63	n.d.	n.d.	4.74	n.d.

a The geometric mean and geometric standard deviation are shown for each assay. A negative log 10 reduction value indicates an increase in microbial biomass or activity. Log_10_ reductions that were different from zero with statistical significance based on Student’s *t*-test are indicated by an asterisk. Cumulative log _10_ reduction values are shown for only BAC filtrate or RO permeate, which were calculated using only samples for which data from all five assays were available (*n* = 4). n.d. = not determined.

In the tertiary wastewater, cell counts were high (approximately 10^6^–10^7^ cells/mL) with relatively low variability (Table 1) and were similar to previous reports for secondary wastewater.^29,48^ Consistent with the cellular membrane being the primary site of damage by ozone,^49,50^ reductions of cell counts and intracellular ATP by ozone (approximately 2.2 log_10_) were significantly greater than total ATP (0.73 log_10_) (*p* < 0.001; ANOVA). Interestingly, total ATP was largely unchanged by BAC filtration (Table 1), possibly due to the conversion of extracellular ATP into intracellular ATP by the microbial community in the BAC filter.^49^ Cell counts and ATP exhibited low variation in the BAC filtrate throughout the three months of sampling (Figure 3a), indicating that the filters were operating at a steady state with respect to microbial shedding.

Cell counts in the MF/UF permeate (Table 1, Figure 3a) were approximately 1 log_10_ higher than those previously reported,^29,48^ despite the MF/UF reducing turbidity to low values (average BAC effluent and MF/UF filtrate turbidities of 0.17 and 0.03 NTU, respectively) as expected. In turn, average log_10_ reductions for total and intact cell counts by MF/UF (1.21 and 1.32 log_10_, respectively) were 3 log_10_ lower than those we previously observed at a potable reuse facility in El Paso, TX (4.60 and 4.28 log_10_, respectively);^29^ notably, chloramine disinfectant was applied upstream of MF/UF in El Paso, whereas no chloramines were applied here during the study period. Based on flow cytometry data, there was a significantly higher proportion (*p* < 0.001, Student’s *t*-test) of “high nucleic acid” content bacteria^39^ in the MF/UF permeate (82 ± 13%) as compared to the BAC filtrate (51 ± 10%) (Figure S2), which is indicative of recent growth. There were no significant differences in log_10_ reductions by MF/UF among total cell counts, intact cell counts, total ATP, and intracellular ATP (*p* < 0.001, ANOVA), which was unexpected based on previously reported low log_10_ reductions for total ATP by MF/UF.^29^

Similar to the MF/UF filtrate, total and intact cell counts in the RO permeate (Table 1, Figure 3a) were approximately 1 log_10_ higher than those previously reported,^2,29^ and a high fraction of high nucleic acid bacteria was also observed (75 ± 15%) (Figure S2). The log_10_ reductions measured for total ATP by RO (1.82 log_10_) were lower than previously reported (up to 3 log_10_),^29,51^ likely because the ATP concentrations in the RO feed were already low, and the concentrations in the permeate were close to the detection limit. There were no significant differences in log_10_ reductions by RO among the four methods for cell counts and ATP (*p* < 0.001, ANOVA). For the AOP, the reduction of intact cell counts (≥0.86 log_10_) was higher than that for total cell counts (0.39 log_10_), which is consistent with previous reports that the fraction of damaged cells increased due to the exposure to free chlorine^52,53^ and in contrast with a previous study on UV-H_2_O_2_ treatment.^29^

Results for 16S rRNA gene copies were compared to cell counts and ATP at treatment steps, where all methods were used (tertiary wastewater, BAC filtrate, and RO permeate). Cumulative log_10_ reductions for each method were calculated (Table 1) using only results from sampling days for which data from all five assays were available (*n* = 4). Cumulative log_10_ reductions at RO permeate for the 16S rRNA gene were significantly higher as compared to reductions for total ATP (*p* = 0.03); all other comparisons among the five methods were not significant (*p* < 0.001; ANOVA with post-hoc Tukey test). Lastly, higher geometric standard deviations were observed for 16S rRNA gene copy measurements as compared to cell counts or ATP at every sampling location (Table 1). Unsurprisingly, these results reflect that measuring 16S rRNA gene copies via qPCR, which is indirect and follows concentration and DNA extraction, is less precise than directly measuring cell counts via flow cytometry or ATP analysis.

### 3.4 Evaluation of Microbial Recovery Using Dead-End Ultrafiltration

Recovery of microorganisms from ultrapure water (e.g., RO permeate) can require a concentration of large quantities of water to distinguish the true microbial signal from noise and contamination. We used dead-end ultrafiltration, followed by PEG flocculation, to concentrate bacteria, resistance genes, and viruses. It was expected that recovery would differ for the different sample matrixes. Therefore, we assessed the recovery efficiency of primary (i.e., dead-end ultrafiltration) and secondary (i.e., PEG flocculation and centrifugation) concentration for recovering total cell counts at each sampling location. An independent evaluation of virus recovery was not feasible due to logistical constraints.

Cell recovery efficiencies varied widely for primary concentration (i.e., dead-end ultrafiltration; 1.5–259%) but less so for secondary concentration (i.e., PEG precipitation; 4.2–30%) (Figure S4). Geometric mean cell recovery efficiencies for primary concentration (Table S12) were high for samples of the tertiary wastewater (71%, *n* = 3), BAC filtrate (104%, *n* = 4), and MF/UF storage tank (85%, *n* = 2). These recoveries are similar to but more variable than recoveries previously reported for surface and tap waters using similar but not directly comparable ultrafiltration methods (Table S12).^4,5,15^ Recoveries were relatively low for RO samples for both primary (14.5%, *n* = 10) and secondary concentration (14.1%, *n* = 8), resulting in an overall recovery efficiency (i.e., through both primary and secondary concentration) of 2.18% for RO permeate (*n* = 8). Low recovery for primary concentration may be due to incomplete recovery of bacteria that sorbed to the dead-end ultrafilter membrane, whereas low recovery for secondary concentration may be due to poor flocculation or centrifugation of bacteria in low-particulate water like RO permeate or incomplete re-homogenization of the floc pellet after centrifugation. It is unlikely that qPCR inhibition caused the low observed 16S rRNA gene concentrations because qPCR inhibition was not observed in testing (Table S6).

**Figure 4:**
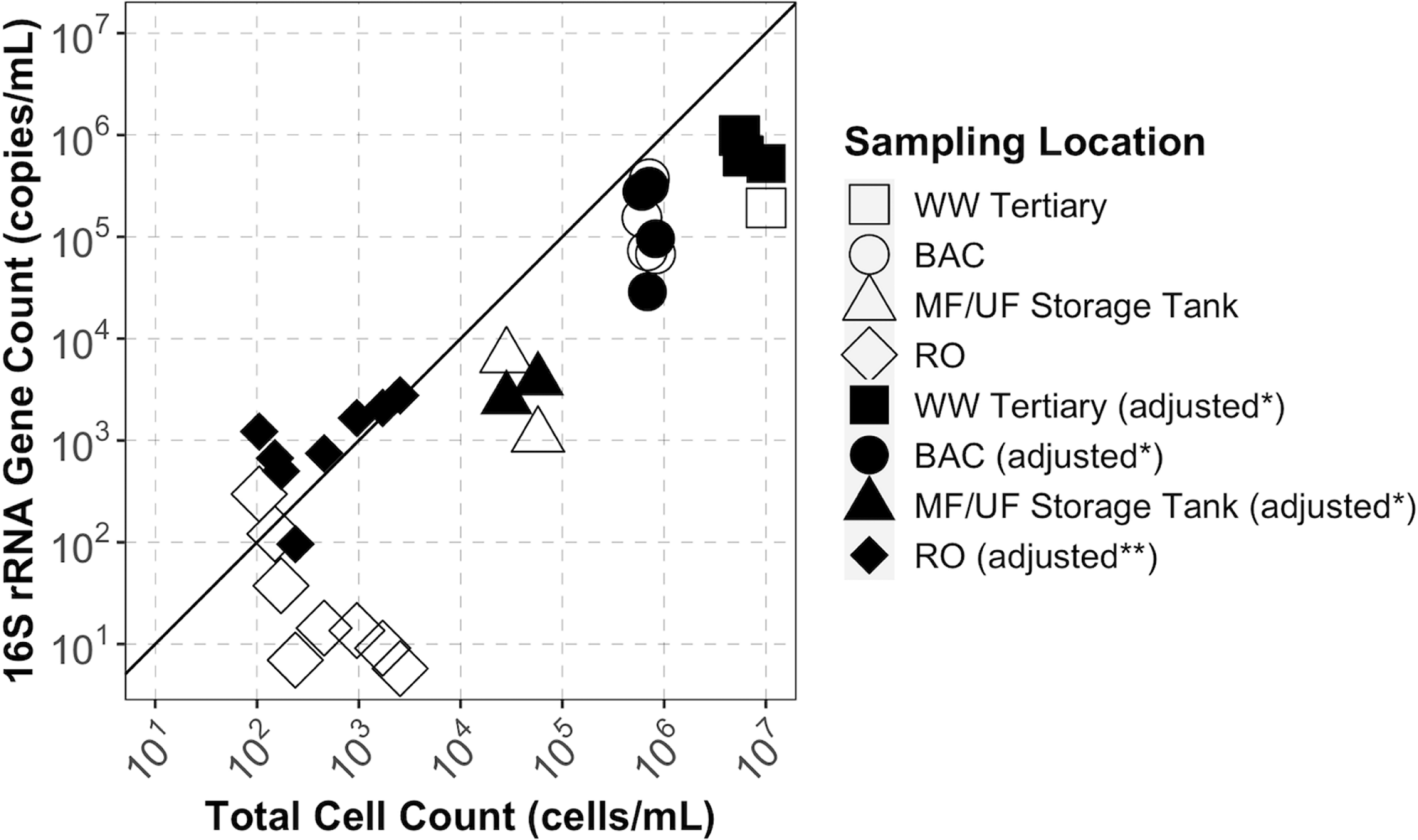
Scatterplot of the 16S rRNA gene count and total cell count in all bulk water samples from the advanced treatment train. All samples were analyzed in technical triplicate by both qPCR and flow cytometry. For visual reference, a 1:1 correlation is shown by a solid, black line. Sample points where results are not adjusted for recovery are shown as open shapes. Sample points where qPCR results for the 16S rRNA gene were adjusted to account for recovery are shown as solid fill. For recovery-adjusted results, *16S rRNA gene concentrations for samples of tertiary wastewater, BAC, and MF/UF storage tank were adjusted for primary recovery only, but **samples of RO permeate were adjusted for both primary and secondary recovery. Two tertiary wastewater samples had >99% recovery by the primary concentration, and the nonadjusted points are hidden behind adjusted points.

The measured concentrations of the 16S rRNA gene were significantly lower than the total cell count in all samples before adjusting for the recovery efficiency (*n* = 17; *p* <0.001, unpaired two-sample Wilcoxon test; Figure 4). After adjusting the RO permeate samples for the recovery efficiency to account for losses of cells during primary and secondary concentration steps, the ratio of the 16S rRNA gene to total cell counts was slightly greater than 1:1 in most samples (Figure 4), which is what we would expect given that there is generally more than one copy of 16S rRNA gene per bacterium.^54^ For samples of tertiary wastewater, BAC filtrate, and MF/UF storage tank, we were able to estimate the recovery efficiency only for the primary concentration step due to experimental constraints. For these sample types, the adjusted results for the 16S rRNA gene remain approximately 1 log_10_ lower than total cell counts, which suggests that a substantial loss of cells occurred during secondary recovery. Other than for results presented in Figure 4, results in this study were not adjusted for estimated recoveries because recoveries varied widely (Table S12) and recovery was not estimated for all samples.

Given the challenges measuring microbial biomass in highly purified sample types, it is important to note that the geometric mean 16S rRNA gene counts (i.e., copies per reaction) were significantly greater in RO permeate (7.00 × 10^5^ copies) than in field blanks (1.58 × 10^5^ copies; *p* = 0.006, Wilcox) and qPCR negative controls (4.61 × 10^4^ copies; *p* < 0.001, Wilcox) (Figure S5), indicating that DNA in samples originated from the sampling source and not from contamination. These results emphasize the importance of using controls to distinguish the sample signal from contamination, especially for low biomass samples, in which contaminant DNA from extraction kits and other laboratory reagents may be of similar magnitude as the sample.^55^

## 4 Discussion

### 4.1 Enteric Viruses

Human adenovirus and JC polyomavirus are pathogenic human viruses previously reported to be present in high concentrations in treated wastewater.^12,13^ We detected adenovirus and polyomavirus in tertiary wastewater and only polyomavirus virus in ozone/BAC effluent. However, we detected neither virus in MF/UF or RO effluents, despite our efforts to concentrate large volumes of water (e.g., up to 4000 L of RO permeate). This finding is consistent with expectations based on the starting concentrations and typical log_10_ reduction values for MF/UF^57^ and RO.^56^ Nonetheless, directly measuring these endogenous pathogens (i.e., they were not seeded) throughout a full-scale advanced wastewater treatment demonstration facility using the high-volume concentration is a novel contribution and provides valuable data regarding the presence and removal of actual human pathogens by advanced treatment processes.

The concentrations of viruses in tertiary wastewater observed here are comparable to those reported from the chlorinated secondary treated wastewater effluent from six wastewater facilities in Japan^45^ and a tertiary treatment facility in the USA^7^ but are approximately 1 to 2 log_10_ lower than values reported for secondary or tertiary effluents in other studies.^46,47^ These studies estimated viral recoveries using surrogate or target viruses but reported virus concentrations uncorrected for recovery efficiencies.

For the combination of ozone and BAC, we observed lower than expected (based on the previous work^56^) log_10_ reductions for human adenovirus and JC polyomavirus. However, we note that the adenovirus reduction by ozone/BAC reported here is a minimum value because some BAC filtrate samples did not amplify above the detection limit. At the average ozone CT of 2.68 mg min/L (average applied concentration of 7.64 mg/L), greater than 6 log_10_ of virus reduction would be expected.^56,58^ However, a previous study of secondary wastewater with a similarly applied ozone concentration (approximately 7 mg/L) also reported relatively low reductions of adenovirus (0.35–1.04 log_10_).^59^ Furthermore, previous studies have reported low reduction rates of viruses by granular media filtration in water reuse for adenovirus (<0.5 log_10_),^7^ and virus reduction by filtration is expected to be low in the absence of coagulation.^60^ For ozone, viruses are inactivated by destruction of the protein capsid and genomic material,^61^ but the qPCR quantification method used herein targeted a short sequence of the viral genome (∼100 bp) that could have been associated with inactive virus.

The measured reduction of polyomavirus by MF/UF (average of >2.0 log_10_) falls within the range of removals previously reported for MF (0.7–4.6 log_10_) and UF (0.5–5.9 log_10_).^57^ Reduction of viruses by MF/UF is typically achieved by several removal mechanisms, including size exclusion, adsorption onto the clean membrane or membrane cake layer, predation, and filtration of particle-associated viruses.^57,62^ In contrast, it is believed that RO membranes primarily reject viruses via size exclusion as the transport through RO membranes is by diffusion and not advection, and the virus passage is attributed to breaches in membrane integrity.^63^ Strategies to detect failure of these diverse reduction mechanisms by membrane treatment is still an area of active research.^63^

The virus concentrations measured here may have been impacted by low recoveries of viruses by the sample concentration methods, but this cannot be confirmed because virus recovery was not evaluated directly. All bulk water samples were processed by dead-end ultrafiltration, polyethylene glycol precipitation, and DNA extraction methods that were optimized for 16S rRNA gene and metagenomic sequencing analyses (manuscript in preparation) but were not optimized for recovery of viral DNA. Large ranges of recovery have been reported for the recovery of adenovirus (1–70%)^7,64,65^ and polyomavirus (33–100%)^66,67^ from surface water, tap water, and diluted raw wastewater using dead-end or tangential ultrafiltration with different secondary concentration methods.

### 4.2 Antibiotic Resistance Genes

Overall, the concentrations and log_10_ reduction values for ARGs reported here align with previous studies. While *sul1* concentrations in tertiary wastewater were comparable to values reported for other secondary/tertiary effluents,^21^ concentrations in RO permeate samples here were similar to or lower than *sul1* concentrations reported for finished and distributed conventional drinking waters (10^–1^ to 10^1^ copies/mL).^3,68,69^ The cumulative log_10_ reduction of *sul1* by RO was similar to that reported for a swine wastewater treatment facility in China that also utilized RO treatment (approximately 4–5 log_10_).^27^ The successful quantification of *sul1* in RO permeate provides further evidence that *sul1* may be a useful surrogate for monitoring antibiotic resistance in potable reuse systems, as previously suggested.^21,25^ In contrast, *bla*_*TEM*_ was reduced to below the limit of detection by MF/UF and, therefore, provided no accurate removal information for membrane treatment. Low concentrations of *bla*_*TEM*_ have previously been reported in conventionally treated wastewater effluents,^70^ indicating the limited utility of using *bla*_*TEM*_ as a surrogate for antibiotic resistance.

### 4.3 Use of Multiple, Complementary Methods for Process Insights and Potential for Online Monitoring

Overall, cell counts and ATP, both of which have been previously proposed as tools for online monitoring, provided quantitative information on the reduction and/or inactivation of microbial cells at every treatment step, with a few notable differences. Cell counts and intracellular ATP were more responsive surrogates than total ATP for ozone and BAC treatment. For ozone, reductions of intracellular ATP and cell counts were >1 log_10_ higher than the reduction for total ATP (Table 1 and Figure S2), likely due to the conversion of intracellular ATP to extracellular ATP from damage to cellular membranes.^49,50^ In contrast, total ATP was expected to increase through the BAC filter due to microbial growth in the filters; however, it appears that extracellular ATP was converted into cell-bound biomass.^49^ Lastly, cell counts and total ATP were reduced through AOP treatment, but intracellular ATP was below the limit of detection in the AOP effluent.

The equivalent log_10_ reduction values reported here for total and intact cells by ozone (2.23 and 2.24 log_10_, respectively; Table 1 and Figure S2) were surprising, given previous observations of higher log_10_ reductions for intact as compared to total cell counts by ozone treatment.^29,71^ The log_10_ reductions measured here were significantly greater than those we previously observed through ozone at a pilot potable reuse facility in El Paso, Texas (0.20 and 0.91 log_10_, respectively, *p* = 0.002 and <0.001, respectively).^29^ This difference was likely driven by a larger applied ozone concentration in the present study site (average = 8.17 mg/L) compared to El Paso (∼3.5 mg/L). Calculation of ozone CT (i.e., mg-min/L) would improve this comparison; the ozone CT here was an average of 2.67 mg min/L, but ozone CT was not able to be calculated at El Paso.

The low reductions of cell counts by MF/UF observed here (∼1 log_10_) were also surprising, given previous reports of reductions exceeding 4 log_10_ for MF/UF.^29,72^ The lower reductions here corresponded to higher cell counts in the MF/UF filtrate (Table 1 and Figure 3b) than previously reported in MF/UF effluents.^29,72^ Membrane defects likely did not drive high filtrate cell counts in the MF/UF effluent here; a review of performance data indicated that the membranes were granted 4 log_10_ reduction^3^ for *Giardia* cysts and *Cryptosporidium* oocysts based on daily pressure decay tests, and fecal and total coliform bacteria rejection was greater than 3 log_10_. Rather, it is likely that microbial growth occurred downstream of MF/UF in the absence of a chloramine residual,^73^ which is not representative of conventional practice for control of biofilms in membrane systems.^1^ This possibility of growth is supported by a significant increase (*p* < 0.001) in the fraction of “high nucleic acid” content bacteria^39^ between the BAC filtrate (51 ± 10%) and MF/UF filtrate (82 ± 13%) (Figure S2). Increases in high nucleic acid bacteria have previously been linked to microbial growth in membrane-treated waters.^49,74^ The possibility of growth is also supported by observations of distinct microbial communities between the BAC filtrate, MF/UF storage tank, and RO permeate (manuscript in preparation).

The reductions of ATP of approximately 1.8 log_10_ by RO were surprisingly low given that previous studies have reported ATP reductions by RO of nearly 3 log_10_.^29,51^ However, our ability to measure ATP removal here may have been limited by low concentrations in the RO feedwater (more than 1 log_10_ lower than those reported for other facilities) and concentrations in the RO permeate that were close to the detection limit. Compared to advanced treatment facilities that directly treat secondary wastewater with MF/UF and RO, ATP concentrations in the RO feedwater here were low, following the treatment via ozone and BAC filtration.^29,51^ Thus, more work is needed that evaluates online monitoring tools at facilities with different treatment trains to characterize the limitations of different monitoring methods.

### 4.4 Recovery of Microbial Biomass using Dead-End Ultrafiltration

The concentration and extraction methods used here yielded sufficient DNA to successfully quantify the 16S rRNA and *sul1* genes in the RO permeate, based on comparisons of postamplification quantities against field blanks and qPCR negative controls (Figure S5). Experimental reagents commonly contain DNA, including DNA extraction kits and even molecular-grade waters and PCR master mixes.^75,76^ Alternative concentration methods have failed to yield sufficient DNA in the RO permeate to distinguish permeate communities from the field or analytical blanks using DNA sequencing.^18,19,77^

Although we expected the 16S rRNA gene concentrations to be higher than cell counts because cells have multiple copies of the 16S rRNA gene,^54^ we observed the opposite in samples with concentrations that were not adjusted for recovery. However, in samples where concentrations were adjusted by the primary and secondary recovery efficiencies using cell count estimates (RO permeate), most 16S rRNA gene concentrations were higher than cell counts, which were measured directly via flow cytometry and thus did not have concentration or DNA extraction losses to consider (Figure 4). Nonetheless, adjusting for cell recovery can be problematic, as we found that recovery was highly variable within even the same sample type and some measured recoveries were greater than 100 percent (Figure S4). The variable recoveries for primary concentration observed here differ from the relatively low variability (standard deviations ranging from 4 to 21 percent) reported in previous studies using dead-end ultrafiltration (without filter blocking) to recover bacteria from surface waters over a range of turbidites (16–92 NTU)^15^ and from tap water.^4^ These studies may have achieved lower variability in their recoveries through the use of an ultrafilter feed with known stable microbial concentrations, whereas the feed used herein may have had varying cell counts over the ultrafiltration sampling collection period. Notably, other studies measured recovery on specific organisms (e.g., *Escherichia coli*, *Clostridium perfringens*, and Enterococci),^4,5,15^ whereas our study measured recovery using total cell counts.

The accuracy and variability of the cell recovery efficiencies were affected by two experimental constraints. First, recovery by the primary concentration was calculated using a single total cell count measurement of bulk water at the start of dead-end ultrafiltration. This single grab sample measurement likely does not represent the mean cell counts over the sampling period because cell counts could have varied over the sample concentration time frames (0.5–48 h). Second, the estimate of recovery by the secondary concentration was likely artificially low due to incomplete dispersion of sample floc after concentration by PEG and centrifugation. Adjusting for recovery prior to calculating log_10_ reductions by treatment processes is not recommended without a comprehensive method analysis. Lastly, because the measurement of recovery efficiency can introduce biases (e.g., for calculation of log_10_ reductions), it is recommended that both corrected and uncorrected values be reported.

## 5 Conclusions

This research addressed two ongoing needs for implementation of advanced treatment trains for potable reuse: monitoring strategies for crediting pathogen reduction and process control. We applied enhanced sampling and analytical techniques that yielded promising results but require further research and application to assess their utility in monitoring advanced treatment trains.

Despite the use of dead-end ultrafiltration and polyethylene glycol precipitation to concentrate high sample volumes (up to 4000 L), adenovirus and polyomavirus concentrations were below the detection limit in MF/UF and RO permeates. However, by concentrating large volumes of water, we were able to demonstrate that the reduction of polyomavirus by advanced treatment was typically greater than 4 log_10_. Quantifying the reduction of enteric viruses throughout advanced treatment is challenging because the target virus concentrations in advanced purified water are far below current detection limits. To meet the tolerable annual risk of infection of 10^–4^ per person per year for drinking water, the enteric virus concentration in advanced purified water must be below 2.2 ×10^–7^ per liter;^3^ therefore, over 10,000,000 L of product water would need to be filtered, not accounting for the recovery efficiency. A promising alternative may be to use high-volume filtration to quantify nonpathogenic viral surrogates, such as pepper mild mottle virus,^11,78^ crAssphage,^11,79^ or Aichi virus.^10^ These viral surrogates can be present in concentrations higher than human viruses in wastewater and have been used to demonstrate high log_10_ reduction values for advanced treatment processes.^6,9^

In contrast with the viruses, the use of dead-end ultrafiltration and polyethylene glycol precipitation enabled reliable quantification of the 16S rRNA gene and *sul1* in all samples, including RO permeate. In the RO permeate, 16S rRNA gene counts were significantly greater than those in field blanks and qPCR negative controls, providing confidence in ongoing 16S rRNA gene sequencing and metagenomic analyses (manuscript in preparation). Furthermore, the novel use of flow cytometry cell counts to estimate recovery efficiency has the potential to be improved via more frequent sampling of cell counts during the sample concentration by ultrafiltration and improving sample floc dispersal (after polyethylene glycol precipitation).

The low concentrations of *sul1* and *bla*_*TEM*_ in the RO permeate support previous conclusions that advanced treatment trains for potable reuse will typically reduce antibiotic resistance to negligible levels, comparable to background concentrations (e.g., conventional drinking water).^3,22^ Additionally, concentrations of *sul1* were above the quantification limit in the RO permeate and were greater than *bla*_*TEM*_ at all sampling locations, supporting previous work that observed *sul1* in greater abundance than numerous other antibiotic resistance genes.^80−83^ Future work could explore the use of *sul1* as a model antibiotic resistance gene for evaluating wastewater-impacted systems.

Lastly, both flow cytometry and ATP provided insights into cellular quantities and viability across every major treatment process, with intact cell counts best capturing changes throughout the treatment. We previously proposed flow cytometry and ATP as online, continuous monitoring tools to demonstrate microbial reduction by MF/UF and RO membranes.^29^ However, lower log_10_ reductions for cell counts and ATP were observed herein for MF/UF and RO than expected. These results provide insights into how treatment train design (e.g., use of chloramine residual upstream of MF/UF) can influence the effectiveness of process monitoring strategies (e.g., measurement of ATP reduction by RO).

## Supporting Information Available

(1) Information on calculation of primary and secondary recovery efficiencies, (2) further details for qPCR methods, and (3) several tables and figures referenced in the manuscript (PDF)
