# Supplementary material for: Microbial Water Quality through a Full-Scale Advanced Wastewater Treatment Demonstration Facility
Source: ACS ES T Eng. 2022 Oct 6;2(12):2206–19. doi: 10.1021/acsestengg.2c00198 (PMC9745798; doi:10.1021/acsestengg.2c00198)
Supplement: Supplementary file 1 — ee2c00198_si_001.pdf [file ee2c00198_si_001.pdf]

Title: Microbial water quality through a full-scale advanced wastewater treatment demonstration facility

Authors: Scott Miller[1,2], Hannah Greenwald[1,2], Lauren C. Kennedy[1,2,3], Rose S. Kantor[1,2], Renjing Jiang[1,2], Aleksey Pisarenko[4], Elise Chen[4], and Kara L. Nelson*[1,2]

[1]Department of Civil and Environmental Engineering, College of Engineering, University of California, Berkeley; Berkeley, CA, 94720, United States
[2]National Science Foundation Engineering Research Center for Re-inventing the Nation's Urban Water Infrastructure (ReNUWIt); Berkeley, CA, 94720, United States
[3]Department of Civil and Environmental Engineering, College of Engineering, Stanford University, Stanford, CA, 94305, United States
[4]Trussell Technologies, Inc.; Solana Beach, CA, 92075, United States

*Corresponding author email: karanelson@berkeley.edu

Table of Contents

1. SUPPLEMENTARY METHODS 2
   1.1. CALCULATION OF RECOVERY EFFICIENCIES BY PRIMARY AND SECONDARY CONCENTRATION 2
   1.2. ADDITIONAL QUANTITATIVE PCR METHODS 2
2. SUPPLEMENTARY TABLES 3
3. SUPPLEMENTARY FIGURES 14
4. AUTHOR CONTRIBUTIONS 18
5. REFERENCES 19

1. Supplementary Methods

1.1. Calculation of Recovery Efficiencies by Primary and Secondary Concentration

Recovery efficiencies for primary (dead-end ultrafiltration) and secondary concentration (PEG flocculation and centrifugation) were calculated as follows:

$$Primary\ Recovery\ Efficiency = \frac{TCC_{Backflush} * V_{Backflush}}{TCC_{Grab\ Sample} * V_{Ultrafilter\ Feed}}$$

$$Secondary\ Recovery\ Efficiency = \frac{TCC_{Resuspended\ Floc} * V_{Floc\ Volume}}{TCC_{Backflush} * V_{Backflush}}$$

Where:

- $TCC_{Backflush}$ = total cell counts in ultrafilter backflush water
- $V_{Backflush}$ = volume of the ultrafilter backflush water
- $TCC_{Grab\ sample}$ = total cell counts in the raw grab sample
- $V_{Ultrafilter\ feed}$ = volume of water filtered by the ultrafilter
- $TCC_{Resuspended\ floc}$ = total cell counts in the resuspended RO permeate floc
- $V_{Floc\ volume}$ = volume of the resuspended RO permeate floc

1.2. Additional Quantitative PCR Methods

For qPCR inhibition analysis, we used undiluted samples with less than 100 ng of DNA per qPCR assay well. Results of inhibition testing indicated that samples with less than 100 ng of DNA per qPCR assay well were uninhibited; therefore, sample DNA were diluted to ensure less than 100 ng of DNA was added to each qPCR well. Dilution of sample DNA into qPCR wells varied from no dilution to 100-fold dilution. Of the 242 samples analyzed among all of the qPCR assays, one sample was diluted 100-fold, four samples were diluted 20-fold, and 16 samples were diluted 10-fold; all other samples were diluted 5-fold or less.

On the StepOnePlus software, we applied the same threshold to all samples within an assay. The threshold was selected based on average threshold values determined by the instrument and checked visually to best cross the linear portions of every standard and sample amplification curve. **Table S2** shows the thresholds chosen for each assay.

For all samples with mean gene counts above their respective assay LOD, all three replicates amplified above the respective LOD. No sample data were removed from data analysis.

gBlock™ standards were prepared as follows: probes were prepared as 100 nm PrimeTime 5’ 6-FAM/ZEN/3’ IBFQ (16S rRNA) or PrimeTime Eco 5’ 6-FAM/ZEN/3’ IBFQ (adenovirus and polyomavirus) purified by HPLC. Primers for all assays were prepared as gBlocks™ Gene Fragments, RxnReady® Primer Pool – Oligo Mix Products purified by standard desalting.

2. Supplementary Tables

Table S1: Sample dates and times for dead-end ultrafiltration (for DNA and qPCR analysis), and grab samples for flow cytometry and ATP. Times are shown in 24-hour format. Dead-end ultrafiltration sampling for RO permeate was conducted overnight to maximize quantity of water filtered due to low microbial quantities in RO permeate.

Date	Type of Sample	Tertiary	Ozone	BAC	MF/UF	MF/UF Storage Tank	RO	AOP
September 17, 2017	Dead-end Ultrafiltration	10:15-10:37		9:10-10:54			9:00-11:00 (overnight)	
	Grab	10:38	9:29	10:55	9:50	9:50	10:50	10:25
September 21, 2017	Dead-end Ultrafiltration						15:30-11:55 (overnight)	
	Grab						15:30	
September 26, 2017	Dead-end Ultrafiltration	14:20-time unknown		14:00-time unknown			14:00-11:15 (overnight)	
	Grab	14:20	14:00	14:00	13:30	13:15	14:00	14:15
October 10, 2017	Dead-end Ultrafiltration	15:30-time unknown		15:14-time unknown		16:45-time unknown	17:00-time unknown (overnight)	
	Grab	15:24	16:10	15:14	16:45	16:45	17:00	16:30
November 7, 2017	Dead-end Ultrafiltration							
	Grab	9:45	9:45	9:45	10:00*	9:50	10:05	10:00
November 14, 2017	Dead-end Ultrafiltration	11:15-11:35		10:50-12:30		11:35-13:25	11:30-time unknown (overnight)	
	Grab	11:15	11:55	10:50	11:35	11:05	11:30	11:30
December 14, 2017	Dead-end Ultrafiltration	14:30-14:55		14:00-15:25		14:25-16:45	15:30-time unknown (overnight)	
	Grab	14:30	15:25	14:00	15:05	14:25	15:30	15:35
TOTAL	Ultrafiltration	5	0	5	0	3	6	0
	Grab	6	6	6	6	6	7	6

Table S2: Summary table of gBlocks™ standards. Note that additional qPCR targets used in unrelated research studies are included on the gBlocks and are included here to inform characteristics (e.g., length) of each standard.

	Standard A	Standard B	Standard C
Includes F and R Primers for what Targets?	16S rRNA Gene, Legionella pneumophila, Adenovirus, Polyomavirus	Acanthamoeba spp., Vermamoeba vermiformis, sul1, MAC	amoA, blatem
Length	740	997	748
Melting temp (degrees Celsius)	80.6	80.4	78.1
GC Content	0.5243	0.5145	0.4626
Pass IDT complexity screening?	yes	yes	yes
Primer sequences in standard verified?	yes	yes	yes

Table S3: Summary statistics for qPCR standard curves, including amplification efficiency, R^2, and number of replicates amplifying at low end of curve.

	**16S rRNA Gene**	**Human Adenovirus**	***bla* $_{TEM}$**	**JC Polyomavirus**	***sul1***
# of Standard Curves Run =	10	3	7	2	6
Threshold applied	0.14	0.08	0.4	0.14	0.4
Linear range (gene copies per qPCR well)	10^3 - 10^9	10^1 - 10^7	10^1 - 10^6	10^1 - 10^7	10^1 - 10^6
**Amplification Efficiency**					
Arithmetic mean	84.4	87.1	81.2	93.0	83.8
Standard deviation	7.0	8.1	3.9	1.8	6.6
Maximum	93.6	94.7	87.7	94.2	92.7
Minimum	73.3	78.6	77.3	91.8	73.9
**R^2 Values**					
Mean	0.996	0.998	0.998	0.999	0.998
St. Dev.	0.004	0.000	0.001	0.001	0.001
**# of Replicates Amplifying >LoD at Low End of Curve (i.e., 1000 copies for 16S, 10 copies for all other assays)**					
Arithmetic mean	3	2.3	2.4	3	2.7
Minimum	3	2	2	3	2
Maximum	3	3	3	3	3
% of All Replicates that Amplified	100	78	81	100	89

Table S4: List of all qPCR standard curve results. Each plate had one standard curve run on it with three replicates at each 10-fold dilution step of the standard curve. None of the qPCR negative controls for 16S rRNA gene amplified within the linear range of the standard curve. *LoD = limit of detection as defined in the main manuscript methods. **qPCR negative controls were nuclease free water as defined in the methods. ***Standard was accidentally not added to the 1000 gene copy wells (the LoD) for the 16S rRNA gene standard curve on 3/9/19.

Plate #	Date Run	Assay	Effic-iency	Slope	Y-intercept	R^2	Number of Standard Curve Replicates that Amplified at the LoD*	Number of qPCR Negative Control** Replicates that Amplified	Avg C_q at LoD	Standard Deviation of C_q at LoD
105	2/9/19	blatem	87.65	-3.658	40.425	0.999	3	0	36.78	0.25
106	2/18/19	sul1	92.65	-3.511	40.214	0.998	3	0	36.75	0.57
107	2/18/19	16S	91.05	-3.557	41.728	0.999	3	3	31.22	0.06
108	2/18/19	16S	84.9	-3.746	42.534	0.998	3	3	31.3	0.26
113	2/21/19	sul1	87.13	-3.674	40.727	0.998	3	0	37.33	0.68
114	2/21/19	blatem	77.36	-4.018	42.848	0.998	2	0	38.39	0.46
115	2/24/19	16S	88.33	-3.637	41.518	0.998	3	3	30.69	0.08
120	2/26/19	sul1	82.47	-3.829	42.021	1	2	0	37.96	0.10
121	2/26/19	blatem	79.6	-3.932	42.741	0.998	2	0	38.32	0.74
122	2/26/19	16S	78.48	-3.975	44.065	0.996	3	3	32.85	0.16
125	2/27/19	sul1	77.06	-4.03	42.920	0.998	2	0	39.17	1.18
126	3/5/19	blatem	80.49	-3.899	42.048	0.996	3	0	37.94	1.04
129	3/9/19	16S	78	-3.994	44.807	0.992	3	3	***	***
130	3/7/19	sul1	88.49	-3.632	39.872	0.999	3	0	36.05	0.22
131	3/7/19	blatem	80.8	-3.889	41.534	0.997	3	0	38.12	0.67
137	3/20/19	16S	77.72	-3.979	45.240	0.994	3	3	33.74	0.06
138	3/12/19	blatem	85.198	-3.736	41.700	0.997	2	0	37.44	0.35
139	3/19/19	sul1	85.034	-3.742	40.899	0.997	3	0	37.44	0.97
140	3/19/19	16S	93.555	-3.487	41.638	0.997	3	3	31.38	0.09
141	3/9/19	adeno-virus	94.682	-3.456	37.822	0.998	3	0	35.14	0.08
142	3/9/19	polyoma-virus	91.75	-3.537	41.196	0.998	3	0	37.37	0.91
143	3/27/19	sul1	73.885	-4.162	44.013	0.999	1	0	39.88	NA
144	3/26/19	blatem	77.3	-4.02	43.184	0.998	2	0	39.16	1.33
145	3/27/19	16S	90.623	-3.569	43.611	0.987	3	3	33.17	0.41
146	3/26/19	adeno-virus	88.09	-3.656	39.594	0.998	2	0	36.02	0.86
147	3/28/19	polyoma-virus	94.234	-3.468	40.680	0.999	3	0	37.21	0.76
148	3/28/19	16S	73.276	-4.189	51.799	0.996	3	3	39.21	0.63
149	4/17/19	adeno-virus	78.6	-3.97	40.898	0.998	2	0	36.7	0.63
151	7/1/19	16S	87.648	-3.658	42.556	1	3	3	31.68	0.08

Table S5: Reaction mixes and thermal cycling conditions for qPCR assays.

Gene Target	Reference Study	Reaction Mix	Reaction Conc. (µM)	Reaction Cycling	Temp (^{o}C)	Time (sec)
**16S universal rRNA gene**	Silkie *et al.* 2009	TaqMan^{TM} Environmental 2.0	(1x)	Pre-denaturation for 95C for 10 min		
		Primer (F and R)	0.9	Denaturation	95	15 s
		Probe	0.25	Annealing	60	30 s
		Bovine serum albumen	0.05	Extension	72	60 s
**Human adenovirus (hexon gene)**	Jothikumar *et al.* 2005	TaqMan^{TM} Environmental 2.0	(1x)	Pre-denaturation for 95C for 10 min		
		Primer (F and R)	0.9	Denaturation	95	15 s
		Probe	0.3	Annealing	55	30 s
		Bovine serum albumen	0.05	Extension	72	60 s
**JC polyomavirus**	Pal *et al.* 2006	TaqMan^{TM} Environmental 2.0	(1x)	Pre-denaturation for 95C for 10 min		
		Primer (F and R)	0.9	Denaturation	95	15 s
		Probe	0.25	Annealing	53	30 s
		Bovine serum albumen	0.05	Extension	72	60 s
blaTEM	Proia *et al.* 2018	PowerUp^{TM} SYBR^{TM} Green	(1x)	UDG activation for 2 min + Pre-denaturation for 95C for 3 min		
		Primer (F and R)	0.3	Denaturation	95	15 s
		Probe	NA	Annealing	60	20 s
		Bovine serum albumen	0.05	Extension	72	60 s
sul1	Proia *et al.* 2018	PowerUp^{TM} SYBR^{TM} Green	(1x)	UDG activation for 2 min + Pre-denaturation for 95C for 3 min		
		Primer (F and R)	0.3	Denaturation	95	15 s
		Probe	NA	Annealing	60	30 s
		Bovine serum albumen	0.05	Extension	72	60 s

Table S6: qPCR inhibition testing results. Samples are not inhibited when the difference between measured delta(Ct) and expected delta(Ct) (see the right-most column) is less than one. Sample dilutions that meet criteria for not being inhibited are shaded green in the right column.

		Expected delta(Ct)			(MEASURED) CT values based on Dilution Factor (x1, 2, 5, etc.)				Measured delta(Ct)			Exp. vs. Meas.		
**Assay**	**Sample Type**	**2x**	**5x**	**10x**	**1x**	**2x**	**5x**	**10x**	**2x**	**5x**	**10x**	**2x**	**5x**	**10x**
blaTEM	Tertiary WW		1.54	2.71		26.67	28.56	29.79		1.89	3.12		0.35	0.41
	RO permeate		1.54	2.71		28.32	30.25	31.36		1.93	3.04		0.39	0.33
sul1	Tertiary WW	1.14	2.65	3.80	22.21	23.16	25.00	26.02	0.95	2.79	3.81	-0.19	0.14	0.01
	RO permeate	1.14	2.65	3.80	19.73	20.74	22.58	23.65	1.01	2.85	3.92	-0.13	0.20	0.12
16S	Tertiary WW		1.49	2.61		18.15	18.88	19.97		0.73	1.82		-0.76	-0.79
	RO permeate	1.12	2.61	3.73	16.91	17.85	19.08	20.24	0.94	2.17	3.33	-0.18	-0.44	-0.40
Adeno-virus	Tertiary WW #1	1.04	1.38	2.42		26.47		29.44			2.97			0.55
	Tertiary WW #2	1.04			30.85	32.12			1.27			0.23		
	BAC	1.04	2.42	3.47		27.29	29.18	30.42		1.89	3.13		-0.53	-0.34
	RO Permeate	1.04	2.42	3.47		29.08	31.23	32.19		2.15	3.11		-0.27	-0.36
Polyoma-virus	Tertiary WW	1.10	2.54	3.64		29.01	30.89	31.91		1.88	2.90		-0.66	-0.74
	BAC	1.10	2.54	3.64						NA	NA			

Table S7: Summary statistics for results from the five qPCR assays. For the calculation of geometric mean ("geomean") and geometric standard deviation ("GeoSD"), all samples with results below the limit of detection ("LoD") were set to the LoD. Prevalence is equal to the total number of samples detected ("Count: Detects") divided by the number of total samples ("Count: Total").

Sampling Location	qPCR Assay	Geomean log_{10} (GC/mL)	GeoSD	Count: Total (n)	Count: Below LoD	Count: Above LoD	Preva-lence (%)
WW Tertiary	16S rRNA gene	6.35E+05	2.10	5	0	5	100
BAC	16S rRNA gene	1.30E+05	1.99	5	0	5	100
MF/UF Storage Tank	16S rRNA gene	2.09E+03	2.71	3	0	3	100
RO	16S rRNA gene	1.88E+01	3.98	10	0	10	100
WW Tertiary	*sul1*	4.59E+03	4.38	5	0	5	100
BAC	*sul1*	1.33E+03	1.34	5	0	5	100
MF/UF Storage Tank	*sul1*	2.52E+02	2.07	3	0	3	100
RO	*sul1*	1.32E-01	2.50	8	0	8	100
WW Tertiary	*blaTEM*	9.74E+01	2.81	5	0	5	100
BAC	*blaTEM*	4.10E+00	17.50	4	0	4	100
MF/UF Storage Tank	*blaTEM*	8.26E-03	1.11	3	2	1	33
RO	*blaTEM*	2.31E-04	2.18	7	7	0	0
WW Tertiary	Adenovirus	2.91E+00	1.99	4	0	4	100
BAC	Adenovirus	3.05E-02	2.30	4	0	4	100
MF/UF Storage Tank	Adenovirus	6.56E-03	1.45	3	3	0	0
RO	Adenovirus	3.71E-04	3.17	9	9	0	0
WW Tertiary	Polyomavirus	1.25E+02	2.08	5	0	5	100
BAC	Polyomavirus	3.05E+00	13.40	5	1	4	80
MF/UF Storage Tank	Polyomavirus	8.26E-03	1.11	3	3	0	0
RO	Polyomavirus	4.32E-04	4.00	7	7	0	0

Table S8: Summary statistics for total and intact cell count measurements across the advanced treatment train. For the calculation of geometric mean ("Geomean) and geometric standard deviation ("GeoSD"), all samples with cell counts below the limit of quantification ("LoQ") were set to the LoQ. Combined results for parallel processes (e.g., "MF/UF" = averaged results for MF and UF) are depicted in the table by grey shading.

Sampling Location	Flow Cytometry Assay	Geomean $\log_{10}$ (cells/mL)	GeoSD	Count (n)	Count (n) less than LoQ	% Less than LoQ
WW Tertiary	Total Cell Count	6.63E+06	1.40	7	0	0
WW Tertiary	Intact Cell Count	4.77E+06	1.42	7	0	0
Ozone	Total Cell Count	4.31E+04	2.93	7	0	0
Ozone	Intact Cell Count	2.89E+04	3.06	7	0	0
BAC	Total Cell Count	6.28E+05	1.23	7	0	0
BAC	Intact Cell Count	5.57E+05	1.20	7	0	0
MF	Total Cell Count	1.55E+04	1.47	7	0	0
MF	Intact Cell Count	1.37E+04	1.40	7	0	0
UF	Total Cell Count	7.23E+04	2.79	7	0	0
UF	Intact Cell Count	4.48E+04	1.98	7	0	0
MF/UF	Total Cell Count	3.90E+04	3.08	14	0	0
MF/UF	Intact Cell Count	2.65E+04	2.26	14	0	0
MF/UF Storage Tank	Total Cell Count	4.95E+04	1.45	7	0	0
MF/UF Storage Tank	Intact Cell Count	4.30E+04	1.37	7	0	0
RO (2-stage)	Total Cell Count	3.52E+02	3.35	7	0	0
RO (2-stage)	Intact Cell Count	2.06E+02	3.27	7	0	0
RO (3-stage)	Total Cell Count	3.55E+02	3.67	7	0	0
RO (3-stage)	Intact Cell Count	2.01E+02	3.68	7	0	0
RO	Total Cell Count	3.53E+02	3.32	14	0	0
RO	Intact Cell Count	2.04E+02	3.28	14	0	0
RO Combined Permeate	Total Cell Count	5.45E+02	2.67	7	0	0
RO Combined Permeate	Intact Cell Count	3.04E+02	2.92	7	0	0
AOP	Total Cell Count	1.61E+02	2.40	6	0	0
AOP	Intact Cell Count	2.95E+01	1.32	6	2	33

Table S9: Summary statistics for total and intracellular ATP measurements across the advanced treatment train. For the calculation of geometric mean ("Geomean") and geometric standard deviation ("GeoSD"), all samples with cell counts below the limit of quantification ("LoQ") were set to the LoQ. Combined results for parallel processes (e.g., "MF/UF" = averaged results for MF and UF) are depicted in the table by grey shading.

Sampling Location	ATP Assay	Geomean $\log_{10}$ (nM)	GeoSD	Count (n)	Count (n) less than LoQ	% Less than LoQ
WW Tertiary	Total ATP	6.81E-01	1.34	5	0	0
WW Tertiary	Intracellular ATP	3.92E-01	1.53	5	0	0
Ozone	Total ATP	1.04E-01	3.52	6	0	0
Ozone	Intracellular ATP	2.54E-03	3.43	6	0	0
BAC	Total ATP	9.51E-02	1.39	6	0	0
BAC	Intracellular ATP	6.98E-02	1.51	6	0	0
MF	Total ATP	3.28E-03	2.33	5	0	0
MF	Intracellular ATP	8.61E-04	2.95	4	0	0
UF	Total ATP	5.90E-03	1.76	5	0	0
UF	Intracellular ATP	3.24E-03	2.08	5	0	0
MF/UF	Total ATP	-2.36E+00	2.11	10	0	0
MF/UF	Intracellular ATP	-2.75E+00	2.98	9	0	0
MF/UF Storage Tank	Total ATP	2.38E+00	2.38	5	0	0
MF/UF Storage Tank	Intracellular ATP	2.06E+00	2.06	5	0	0
RO (2-stage)	Total ATP	1.23E+00	1.23	7	0	0
RO (2-stage)	Intracellular ATP	1.84E+00	1.84	3	0	0
RO (3-stage)	Total ATP	1.63E+00	1.63	6	1	17
RO (3-stage)	Intracellular ATP	3.00E+00	3.01	3	1	33
RO	Total ATP	-3.81E+00	1.42	13	1	8
RO	Intracellular ATP	-4.12E+00	2.25	6	1	17
RO Combined Permeate	Total ATP	-3.51E+00	1.90	5	0	0
RO Combined Permeate	Intracellular ATP	-3.89E+00	2.11	5	0	0
AOP	Total ATP	-4.00E+00	1.00	5	5	100
AOP	Intracellular ATP	NA	NA	NA	NA	NA

Table S10: Log removal values (LRV) for total and intact cell counts by flow cytometry across major treatment processes at the advanced treatment facility. Combined results for parallel processes (e.g., "MF/UF" = averaged results for MF and UF) are depicted in the table by grey shading.

**Sampling Location**	**Total Cell Counts**			**Intact Cell Counts**		
	Average LRV	St. Dev. LRV	Count	Average LRV	St. Dev. LRV	Count
WW Tertiary	0	0	NA	0	0	NA
Ozone	2.23	0.41	5	2.24	0.44	5
BAC	-1.16	0.38	6	-1.28	0.42	6
MF	1.61	0.21	4	1.61	0.19	4
UF	0.94	0.40	6	1.09	0.28	5
MF/UF	1.21	0.48	10	1.32	0.36	9
MF/UF Storage Tank	-0.19	0.39	5	-0.12	0.22	5
RO (2-stage)	2.30	0.81	6	2.24	0.64	5
RO (3-stage)	2.26	0.80	5	2.17	0.65	4
RO	2.28	0.76	11	2.21	0.60	9
RO Combined Permeate	0.04	0.63	4	0.07	0.55	4
AOP	0.39	0.13	5	0.86	0.43	5

Table S11: Log removal values (LRV) for total and intracellular ATP across major treatment processes at the advanced treatment facility. Combined results for parallel processes (e.g., "MF/UF" = averaged results for MF and UF) are depicted in the table by grey shading.

**Sampling Location**	**Total ATP**			**Intracellular ATP**		
	Average LRV	St. Dev. LRV	Count	Average LRV	St. Dev. LRV	Count
WW Tertiary	0	0	NA	0	0	NA
Ozone	0.73	0.48	5	2.20	0.70	5
BAC	0.04	0.55	6	-1.44	0.61	6
MF	1.46	0.27	7	1.92	0.30	7
UF	1.20	0.30	7	1.32	0.42	7
MF/UF	1.33	0.30	10	1.59	0.47	9
MF/UF Tank	-0.34	0.33	5	-0.36	0.39	5
RO (2-stage)	1.87	0.35	7	1.75	0.14	7
RO (3-stage)	1.77	0.66	7	1.89	0.87	7
RO	1.82	0.48	9	1.82	0.51	4
RO Combined Permeate	-0.29	0.30	6	-0.43	0.11	3
AOP	0.44	0.18	4	NA	NA	NA

Table S12. Recovery of total cell count through bulk water primary concentration (ultrafiltration). Results from other studies[1-3] utilizing REXEED 25S filters and similar ultrafiltration methods are shown as points of comparison. These other studies from literature are not directly comparable but are the only studies we found that quantified recovery using ultrafiltration methods.

									Microbial Recovery by Primary Concentration			
**Study**	**Water Type**	**Microbial Target (and if *Seeded)***	**Microbial Quantification Method**	**Type of Ultrafiltration**	**Filter Blocking?**	**Backflushing Solution**	**Water Vol. Collected (L)**	**Turbidity - Average (NTU)**	**Sample Count (n)**	**Range**	**Geo-mean**	**GeoSD**
This Study	Tertiary Wastewat	Total cell count	Flow cytometry	Dead-end	Yes	Refer to methods	30 - 121	NA	3	37 - 100	71.4	1.78
This Study	BAC Effluent	Total cell count	Flow cytometry	Dead-end	Yes	Refer to methods	34 - 336	0.171	4	55 - 253	104	1.96
This Study	MF/UF Storage Ta	Total cell count	Flow cytometry	Dead-end	Yes	Refer to methods	342 - 577	0.03	2	28 - 259	85.3	4.8
This Study	RO Permeate	Total cell count	Flow cytometry	Dead-end	Yes	Refer to methods	1,290 - 3,891	NA	10	1.5 - 102	14.5	3.69
											**Avg**	**StDev**
Smith et al. 2009	Tap ("low turbidity")	*Enterococcus faecalis (seeded)*	mEI agar culture; EPA 1600	Dead-end	No	Tap water	100	0.29	4		93	16
Smith et al. 2009	Tap ("mid turbidity")	*Enterococcus faecalis (seeded)*	mEI agar culture; EPA 1600	Dead-end	No	Tap water	100	1.5	5		71	11
Smith et al. 2009	Tap ("high turbidity")	*Enterococcus faecalis (seeded)*	mEI agar culture; EPA 1600	Dead-end	No	Tap water	100	4.3	6		78	12
Mull et al. 2012	Surface ("low turbidity")	Enterococci	mEI agar culture; EPA 1600	Dead-end	No	Same solution as herein	100	16	3		85	7
Mull et al. 2012	Surface ("mid turbidity")	Enterococci	mEI agar culture; EPA 1600	Dead-end	No	Same solution as herein	100	46	4		73	11
Mull et al. 2012	Surface ("high turbidity")	Enterococci	mEI agar culture; EPA 1600	Dead-end	No	Same solution as herein	100	92	4		86	7
Mull et al. 2012	Surface ("low turbidity")	*Escherichia coli*	mTEC agar culture; EPA 1603	Dead-end	No	Same solution as herein	100	16	3		85	7
Mull et al. 2012	Surface ("mid turbidity")	*Escherichia coli*	mTEC agar culture; EPA 1603	Dead-end	No	Same solution as herein	100	46	4		73	11
Mull et al. 2012	Surface ("high turbidity")	*Escherichia coli*	mTEC agar culture; EPA 1603	Dead-end	No	Same solution as herein	100	92	4		87	7
Kahler et al. 2015	River (Chattahoochee)	*Escherichia coli*	Methods 9222D and 9222G	Tangential-flow	Yes	Same solution as herein	50	5 - 128	4		98	11
Kahler et al. 2015	Lake (Murphy Chandler)	*Escherichia coli*	Methods 9222D and 9222G	Tangential-flow	Yes	Same solution as herein	50	4 - 12	5		85	38
Kahler et al. 2015	Lake (Allatoona)	*Escherichia coli*	Methods 9222D and 9222G	Tangential-flow	Yes	Same solution as herein	50	4 - 5	3		79	12
Kahler et al. 2015	Ground (Jefferson City)	*Escherichia coli*	Methods 9222D and 9222G	Tangential-flow	Yes	Same solution as herein	50	2	4		87	16

3. Supplementary Figures

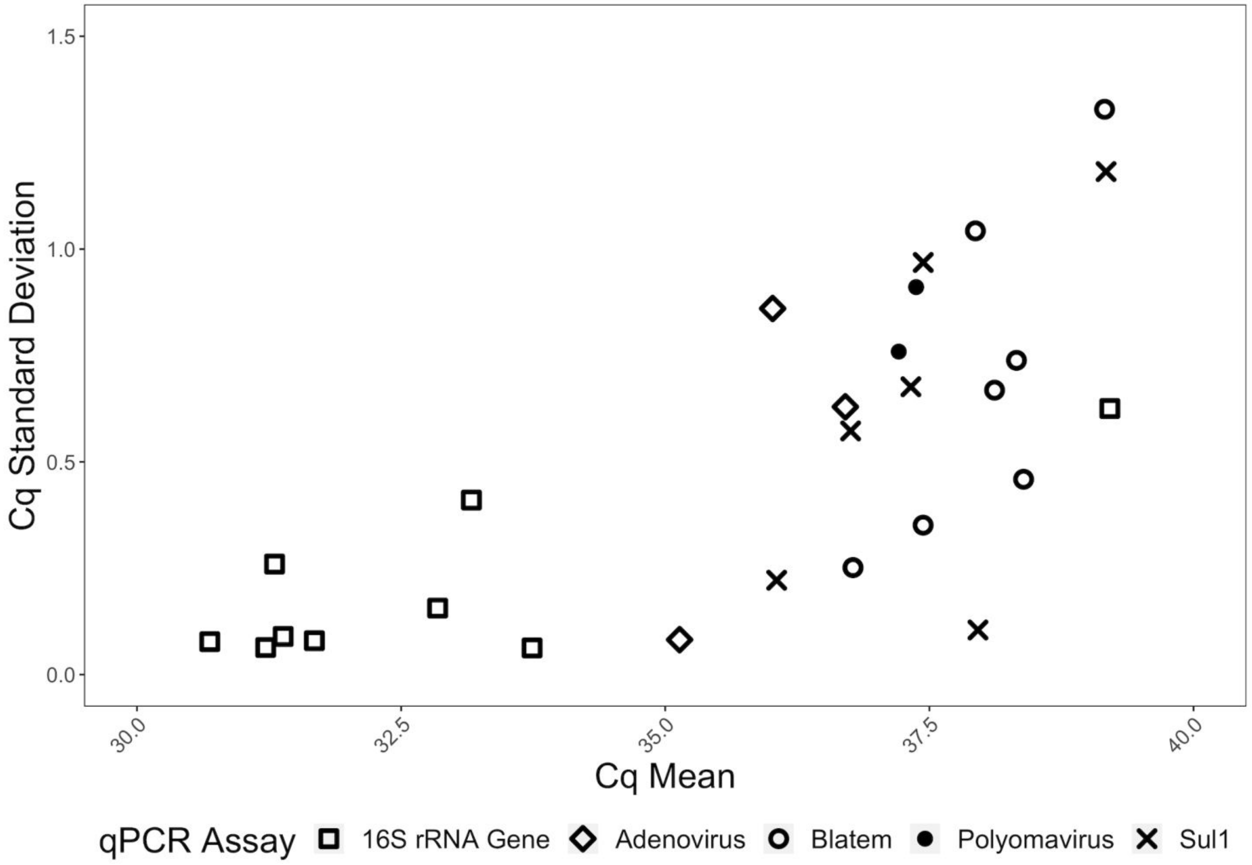

Figure S1. Cq Mean versus standard deviation for standard curve replicates at the respective standard curve LoD (i.e., 1000 gene copies per well for the 16S rRNA gene, and 10 gene copies per well for all other qPCR assays).

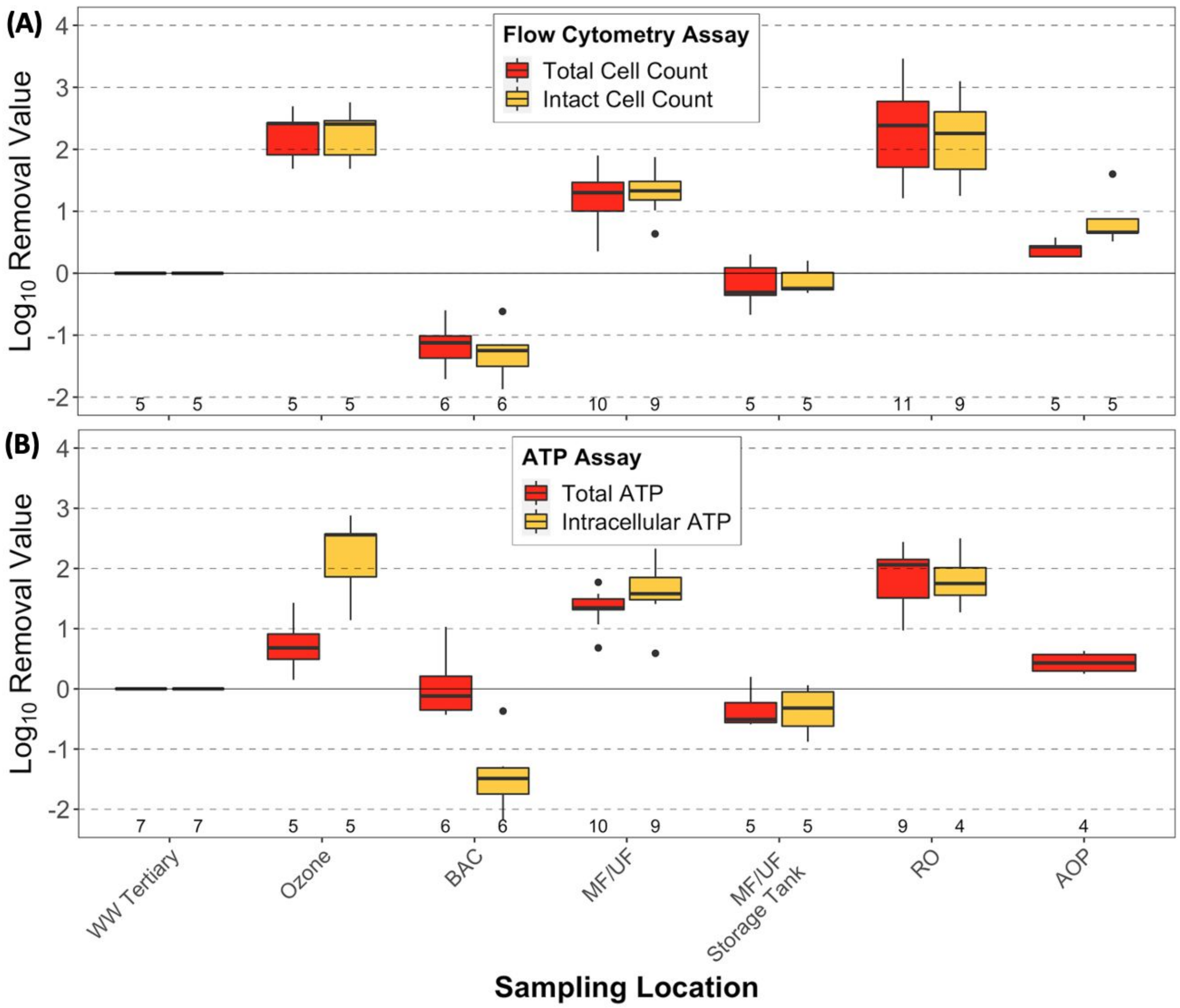

Figure S2. Boxplots of log10 reduction values for (A) total and intact cell counts and (B) total and intracellular ATP throughout the advanced treatment train. Data shown for BAC, MF/UF, and RO are combined measurements from the respective parallel treatment process effluents. The total number of samples taken (n) at each location is located immediately above the x-axis. All samples were analyzed in technical triplicate. Log reduction values were calculated by comparing the microbial abundance in the influent and effluent of each unit process. Intracellular ATP was not below the detection limit for AOP and is not shown here.

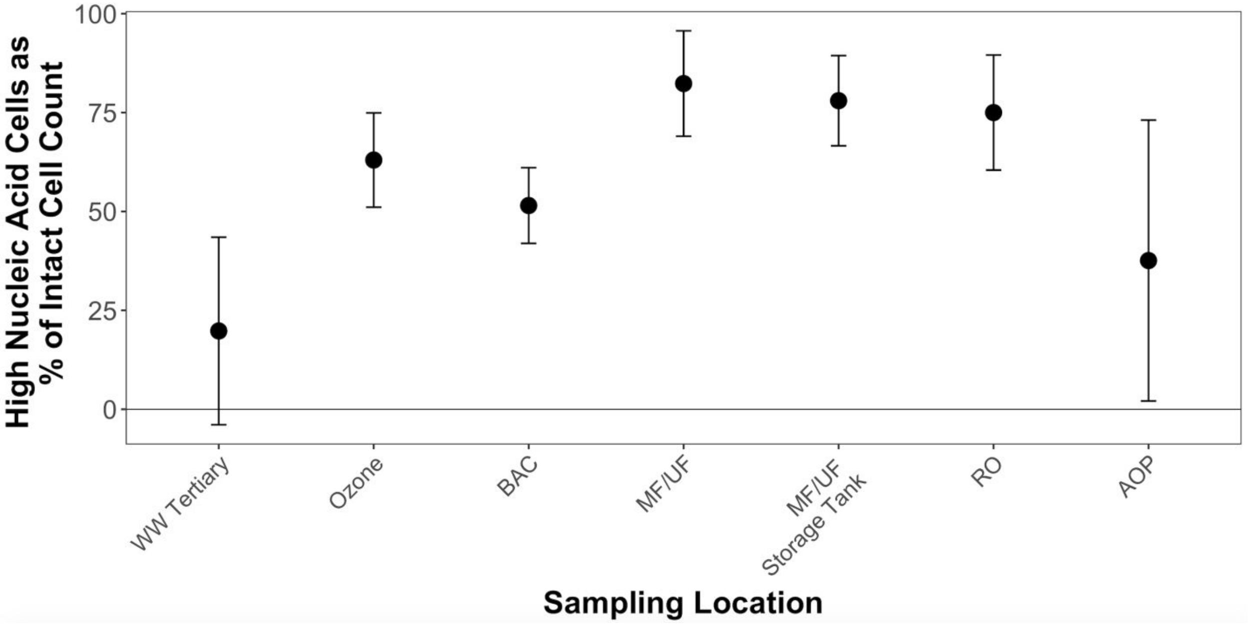

Figure S3. Proportion of intact cell counts that were high nucleic acid bacteria throughout the advanced treatment train. All samples were analyzed in technical triplicates. Error bars represent standard deviation of the average proportion.

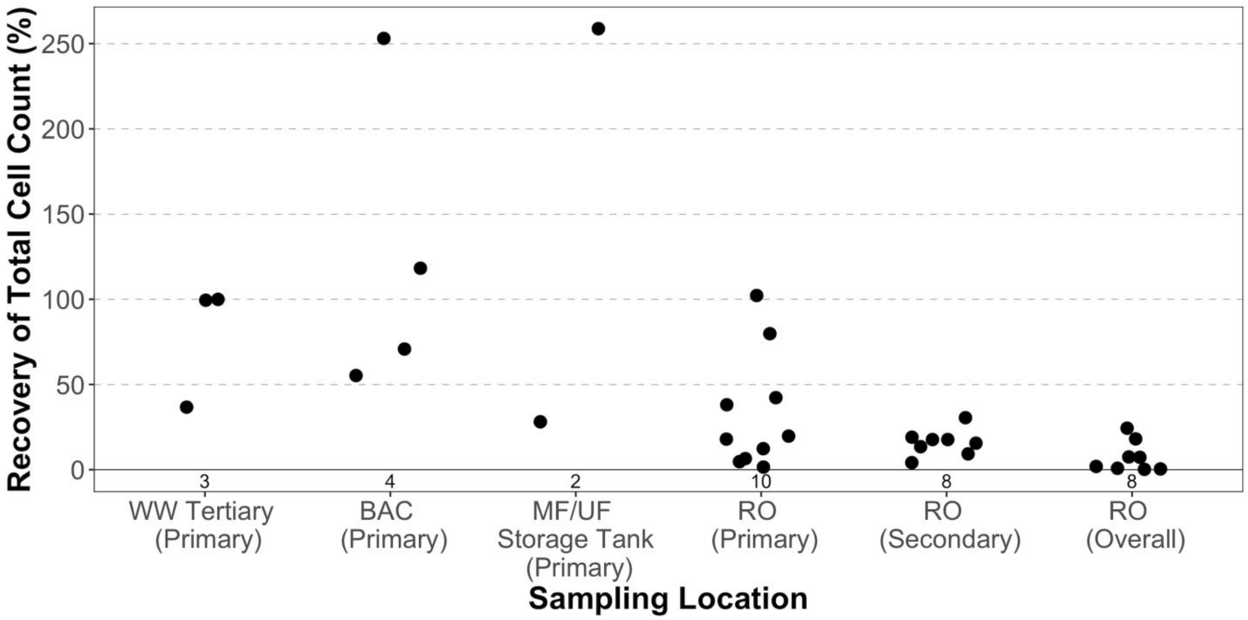

Figure S4: Recovery efficiencies of total cells through primary ("primary"; dead-end ultrafiltration) and secondary concentration ("secondary"; polyethylene glycol flocculation and centrifugation) at sampling locations for which dead-end ultrafiltration was used to concentrate bulk water. Overall recovery was calculated by multiplying primary and secondary concentration efficiencies for respective samples. Secondary recovery was not assessed for tertiary wastewater, BAC, or MF/UF storage tank samples. The total number of samples taken (n) for each type of recovery measurement or calculation is located above the x-axis.

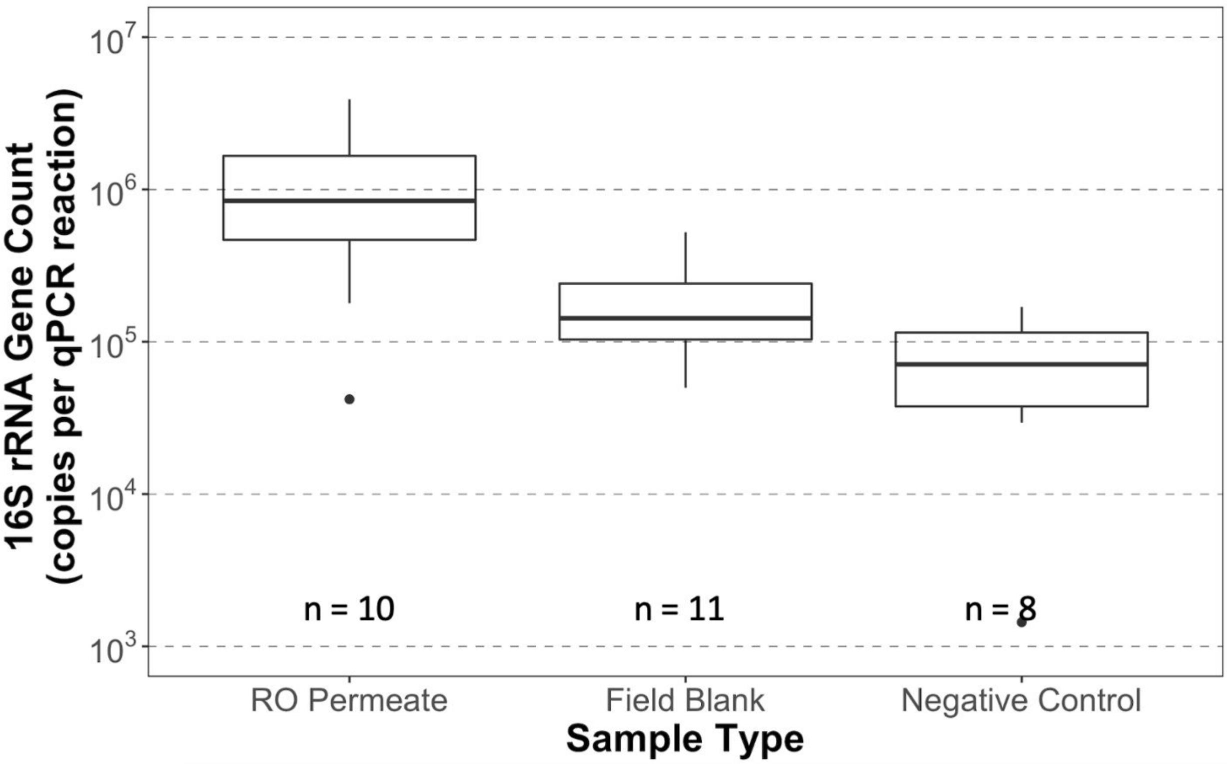

Figure S5: Quantities of the 16S rRNA gene in qPCR reaction wells for RO permeate, dead-end ultrafiltration field blank, and qPCR negative controls (i.e., PCR-grade water). The total number of samples taken (n) at each sampling location is located above the x-axis.

4. Author Contributions

Table of Author Contributions

	UC Berkeley						Trussell Technologies	
	Dr. Scott Miller	Hannah Greenwald	Dr. Lauren Kennedy	Dr. Rose Kantor	Renjing Jiang	Professor Kara Nelson	Dr. Aleks Pisarenko	Elise Chen
Conceptualization	X	X	X	X		X	X	X
Methodology	X	X	X	X	X	X		
Software			X	X				
Validation		X						
Formal Analysis	X							
Investigation	X	X			X		X	X
Resources						X	X	X
Data Curation	X	X	X	X				
Writing - Original Draft	X							
Writing - Review & Editing		X	X	X		X	X	
Visualization	X		X	X				
Supervision				X		X		
Project Administration	X					X	X	X
Funding Acquisition						X		

5. References

1. Smith CM, Hill VR. Dead-End Hollow-Fiber Ultrafiltration for Recovery of Diverse Microbes from Water. *Appl Environ Microbiol*. 2009;75(16):5284-5289. doi:10.1128/AEM.00456-09.

2. Mull B, Hill VR. Recovery of diverse microbes in high turbidity surface water samples using dead-end ultrafiltration. *J Micro Methods*. 2012;91(3):429-433. doi:10.1016/j.mimet.2012.10.001.

3. Kahler A, Johnson T, Hahn D, Narayanan J, Derado G, Hill V. Evaluation of an Ultrafiltration-Based Procedure for Simultaneous Recovery of Diverse Microbes in Source Waters. *Water*. 2015;7(12):1202-1216. doi:10.3390/w7031202.
